# Adipokines in Rheumatoid Arthritis: Emerging Biomarkers and Therapeutic Targets

**DOI:** 10.3390/biomedicines11112998

**Published:** 2023-11-08

**Authors:** Jan Bilski, Agata Schramm-Luc, Marian Szczepanik, Agnieszka Irena Mazur-Biały, Joanna Bonior, Kevin Luc, Klaudia Zawojska, Joanna Szklarczyk

**Affiliations:** 1 Department of Biomechanics and Kinesiology, Chair of Biomedical Sciences, Institute of Physiotherapy, Faculty of Health Sciences, Jagiellonian University Medical College, 31-008 Krakow, Poland; agnieszka.mazur@uj.edu.pl (A.I.M.-B.); klaudia.zawojska@uj.edu.pl (K.Z.); 2 Department of Internal and Agricultural Medicine, Faculty of Medicine, Jagiellonian University Medical College, 31-121 Krakow, Poland; agata.schramm@uj.edu.pl (A.S.-L.); kevin.luc1701@gmail.com (K.L.); 3 Chair of Biomedical Sciences, Institute of Physiotherapy, Faculty of Health Sciences, Jagiellonian University Medical College, 31-034 Krakow, Poland; marian.szczepanik@uj.edu.pl; 4 Department of Medical Physiology, Chair of Biomedical Sciences, Institute of Physiotherapy, Faculty of Health Sciences, Jagiellonian University Medical College, 31-126 Krakow, Poland; joanna.bonior@uj.edu.pl (J.B.); joannam.szklarczyk@uj.edu.pl (J.S.)

**Keywords:** rheumatoid arthritis, adipokines, adipose tissue, skeletal muscle, myokines, inflammation, metabolism, therapeutic targets

## Abstract

Rheumatoid arthritis (RA) is a chronic inflammatory disease manifested by joint involvement, extra-articular manifestations, and general symptoms. Adipose tissue, previously perceived as an inert energy storage organ, has been recognised as a significant contributor to RA pathophysiology. Adipokines modulate immune responses, inflammation, and metabolic pathways in RA. Although most adipokines have a pro-inflammatory and aggravating effect on RA, some could counteract this pathological process. The coexistence of RA and sarcopenic obesity (SO) has gained attention due to its impact on disease severity and outcomes. Sarcopenic obesity further contributes to the inflammatory milieu and metabolic disturbances. Recent research has highlighted the intricate crosstalk between adipose tissue and skeletal muscle, suggesting potential interactions between these tissues in RA. This review summarizes the roles of adipokines in RA, particularly in inflammation, immune modulation, and joint destruction. In addition, it explores the emerging role of adipomyokines, specifically irisin and myostatin, in the pathogenesis of RA and their potential as therapeutic targets. We discuss the therapeutic implications of targeting adipokines and adipomyokines in RA management and highlight the challenges and future directions for research in this field.

## 1. Introduction

### 1.1. Overview of Rheumatoid Arthritis

Rheumatoid arthritis (RA) is a systemic autoimmune disease accompanied by chronic inflammation, leading to joint damage and extra-articular symptoms, which affects women more than men. A characteristic of RA is the presence of synovitis, most often at the level of small and medium joints, leading to symmetrical joint swelling and tenderness. Rheumatoid arthritis tends to fluctuate with periods of flare-ups and remission and, if not well controlled, leads to progressive destruction of the joints [1,2,3,4]. Late diagnosis of RA increases the risk of developing cancer and cardiovascular disease (CVD), reduces life expectancy, and is associated with poorer outcomes [1,2]. Individuals with RA are more likely to have common risk factors associated with CVD, including hypertension, hyperlipidaemia, insulin resistance, and metabolic syndrome, which can be attributed to increased adipose tissue, altered body composition, and elevated levels of pro-inflammatory adipokines [5]. This increased risk for the development of comorbidities, particularly CVD, is one of the most prevalent causes of morbidity and mortality in this patient population [6].

Although the exact aetiology of RA is not yet fully understood, a combination of genetic and environmental factors is believed to play a significant role in its onset [1,2,7]. Rheumatoid arthritis is a chronic immune-mediated disorder in which numerous immune cell types are activated, causing damage predominantly in the joints but also in the vascular system and lungs [1,7,8]. Both Th1 and Th17 lymphocytes play an important role in the pathogenesis of RA. Joint inflammation is the result of an interplay between adaptive and innate immune cells, including T and B lymphocytes, fibroblasts, macrophages, dendritic cells (DC), neutrophils and osteoclasts [2]. Activated autoreactive Th1 and Th17 lymphocytes activate macrophages and fibroblasts in the affected joints via secreted tumour necrosis factor alpha (TNFα), interleukin (IL) 17A, interferon gamma (IFN-γ), and receptor activator of nuclear factor kappa-Β ligand (RANKL). Additionally, autoreactive T lymphocytes support autoreactive B cells in the production of anti-citrullinated protein antibodies (ACPAs) and rheumatoid factor (RF) autoantibodies [7,8]. Rheumatoid arthritis can be subdivided into two main subtypes based on the presence of RF and/or ACPAs [8]. The presence of RF or ACPAs is a poor prognostic factor and is an indication to introduce biological treatment following ineffective initial treatment using a conventional synthetic disease-modifying anti-rheumatic drug (csDMARD) [9].

Several studies have investigated environmental and lifestyle risk factors for RA. Smoking, ozone exposure, and traffic-related air pollution have emerged as significant contributors to RA susceptibility, especially in seropositive patients [10]. Specifically, infections that involve the prevalent periodontal bacterium *Porphyromonas gingivalis* can lead to the initiation of autoimmune responses through the citrullination process, wherein both human and bacterial proteins are modified by protein arginine deiminase (PAD) enzymes within the periodontium [11,12].

The expression of PAD by *P. gingivalis* allows the bacterium to breach local tolerance by converting arginine to citrulline. This breach of tolerance can promote autoimmune responses and the downstream generation of ACPAs [11,12]. It has also been suggested that tobacco smoking predominantly contributes to the progression of rheumatoid arthritis by influencing tissue protein citrullination [13]. Furthermore, there appears to be a potential negative correlation between socioeconomic status and the risk of developing RA. Some studies suggest a potential association between obesity and the development of RA, while others present conflicting evidence, finding no such link [14]. The underlying reasons for these disparities remain elusive, but it is conceivable that various factors such as age, sex, and genetic background may influence the connection between obesity and RA. Notably, the incidence of obesity has experienced a substantial upsurge in recent decades, giving rise to concerns that it may contribute to the heightened prevalence of rheumatoid arthritis [14].

Hormonal factors, microbiome composition, and infectious agents may also contribute to RA development [1,7,10,15,16]. In recent years, an increasing number of studies have explored the intricate relationship between the composition of the gut microbiota and dietary patterns in RA patients. Evidence suggests that dietary factors substantially influence the intricate makeup and dynamics of the human gut microbiota, potentially leading to dysbiosis, which can alter immune regulatory functions and promote a pro-inflammatory state [17,18,19,20].

Environmental factors upregulate the expression of PADs, which can modify peptides by converting arginine to citrulline. After recognition of modified proteins presented by antigen-presenting cells (APCs) such as DCs, T cells support the production of antibodies directed against the altered peptides, including ACPAs. Autoreactive T and B cells initiate an inflammatory cascade in synovial tissues, causing inflammation and damage to the cartilage [21,22]. This leads to synovial enlargement, angiogenesis, osteoclast activation, and bone degradation. Inflammatory cytokines induce the transformation of monocyte–macrophage lineage cells into mature osteoclasts, causing bone resorption and erosion [21].

During these processes, synovial macrophages release pro-inflammatory cytokines such as TNFα, IL-1β, and IL-6, which stimulate the activity of osteoclasts and fibroblast-like synoviocytes (FLS), leading to bone erosion. The synovium, located on the joint capsule’s interior surface, is known to play a crucial role in the pathogenesis of RA. In healthy individuals, the synovium is a thin, delicate tissue layer that lubricates and protects joints. However, in patients with RA, the synovium undergoes inflammation, hyperplasia, and thickening, resulting in synovitis and eventual joint destruction [23]. During the initial stages of RA, the synovium undergoes inflammation due to leukocyte infiltration caused by the activation of endothelial cells expressing adhesion molecules and chemokines [24]. The clinical phase of RA starts with the activation of the adaptive and innate immune systems. Pro-inflammatory cytokines, such as IL-17A produced by Th17 cells and TNFα, IL-1 β, and IL-6 secreted by macrophages, play a crucial role in the pathogenesis of RA [23].

Interleukin-17A is produced mainly by Th17 cells and induces the production of other cytokines, such as IL-6 and TNFα, and chemokines (CCL20, CCL2 and CCL7), which promote the recruitment of neutrophils and monocytes to inflammatory sites in the synovium. These cells release destructive matrix metalloproteinases (MMP), which contribute to joint destruction and damage to surrounding tissues. Matrix metalloproteinase 3 plays a pivotal role in cartilage destruction, while FLS secrete MMP1 and MMP9. Interleukin-17A also promotes the proliferation and survival of FLS, which play a crucial role in bone and cartilage destruction in RA [25]. Moreover, IL-17A induces the production of RANKL by FLS and osteoblasts, leading to the differentiation and activation of osteoclasts, the cells responsible for bone resorption. This process ultimately contributes to bone erosion in RA [25].

Current evidence suggests that FLS are significant players in the development of RA [26] and are crucial in maintaining the homeostasis of synovial fluid (SF) and extracellular matrix (ECM) in healthy joints [27]. When exposed to pro-inflammatory cytokines and chemokines, FLS can become activated and display increased proliferation, migration, and production of inflammatory mediators and enzymes, contributing to joint degradation [23,26]. Rheumatoid arthritis-fibroblast-like synoviocytes play a key role in the development of the RA pannus, a mass of inflammatory, invasive synovial tissue responsible for the erosive damage seen in advanced RA [27]. In RA patients, the interface between bone and pannus is populated with osteoclasts, which have been implicated in marginal joint erosions, as evidenced by various animal models. The rheumatoid synovium is a rich source of osteoclast precursors and factors that stimulate osteoclast activation [28].

Angiogenesis is essential for the expansion of the synovial lining and subsequent invasion of the pannus. Vascular endothelial growth factor (VEGF) is a potent proangiogenic cytokine which plays a central role in the angiogenic process in RA and is produced by various cells within the synovium, including macrophages, FLS, and endothelial cells [29].

Activated FLS in the hyperplastic synovium also mediate cartilage invasion and destruction independently of hyperplastic synovial tissue, which has been attributed to a combination of adhesion-facilitating factors and the production of proteases, particularly MMP3. Adhesion molecules such as intracellular adhesion molecule 1 (ICAM-1) and Vascular Cell Adhesion Molecule 1 (VCAM-1) facilitate the anchoring of RA-FLS to cartilaginous ECM components. Cadherin-11 (CDH-11), expressed by RA-FLS, is relevant in cartilage destruction [30]. Furthermore, FLS can interact with T cells, B cells, and macrophages in the joint, amplifying the inflammatory response [23,26].

Disease-modifying antirheumatic drugs (DMARDs) are essential for the treatment of RA and should be initiated as soon as the diagnosis is made. Conventional synthetic (cs) DMARDs, usually methotrexate, are used in the first line of treatment. If treatment with csDMARDs does not lead to the achievement of the treatment target, biological (b)DMARDs acting via inhibition of TNFα, IL-6, CD20, or inhibiting T cell activation should be introduced. Another therapeutic option is targeted synthetic (ts)DMARDs, acting via inhibition of Janus kinases [9]. Despite the advances made by these treatments in achieving disease remission and preventing joint damage, a significant number of RA patients do not respond adequately to current therapies [7]. Therefore, there is an urgent and unmet need for novel drugs and therapeutic approaches.

### 1.2. Role of Adipose Tissue in RA Pathophysiology

Obesity is a major social issue due to its increasing prevalence and is usually defined as a pathological or excessive accumulation of adipose tissue in the body, which is detrimental to health [31]. Adipose tissue, which is made up of brown adipose tissue (BAT) and white adipose tissue (WAT), plays a pivotal role in metabolism. Brown adipose tissue releases stored energy as heat, whereas WAT stores excess energy as triglycerides [32,33]. Other types of adipose tissue have recently been described [34,35,36]. Moreover, WAT and BAT can regulate metabolism and communicate with other organs through the production of lipokines, adipokines, and batokines, as well as exosomal miRNAs [36,37,38]. The dysfunction of adipose tissue in obesity leads to chronic low-grade inflammation and a shift in adipokine production towards a pro-inflammatory profile [39,40,41] associated with an increased risk of metabolic and cardiovascular diseases and certain malignancies [42,43]. In addition, obesity can contribute to the onset of inflammatory disorders [44,45].

Subcutaneous adipose tissue (SAT) and visceral adipose tissue (VAT), which have different metabolic and immunological characteristics, are the two main anatomical compartments of WAT [46,47]. Accumulation of VAT is strongly associated with an increased risk of metabolic and cardiovascular disorders and mortality [36,48,49,50]. Although SAT may offer some health benefits, visceral fat accumulation, particularly in ectopic obesity, such as perimuscular fat, may have detrimental effects [51,52].

While the primary focus of RA research has traditionally been on the synovium and immune cells, growing evidence highlights the role of adipose tissue in RA pathophysiology. Obesity can lead to mechanical stress on joints, especially weight-bearing joints such as the knees, hips, and feet. In individuals with RA, obesity can exacerbate joint pain and inflammation and contribute to disease progression and joint damage [53]. In the context of obesity in RA, particularly if there is an accumulation of visceral fat, the production of pro-inflammatory adipokines significantly increases the risk of cardiovascular complications [6].

Obesity, particularly visceral obesity, has been identified as a potential risk factor for the development and progression of RA [54,55,56,57,58,59,60,61,62,63,64,65,66], specifically among women [60,63,66]. Recent meta-analyses have provided compelling evidence supporting a positive association between obesity and RA, revealing a dose–response relationship between body mass index (BMI) and RA risk [54,67,68]. This association appears to be more pronounced in women and cases of seronegative disease. However, it is essential to note that the meta-analyses on this subject have presented conflicting results, necessitating further investigation and clarification [54,67,68].

Findings by Li et al. [69] provide valuable insight into the intricate interplay between adipose tissue and the pathogenesis of RA. They studied the role of adipose tissue in inflammatory arthritis by creating fat-free (FF) mice devoid of WAT and BAT but retaining marrow adipocytes. They found that FF mice were resistant to K/BxN serum transfer arthritis. In order to understand the underlying process, the researchers conducted genetic deletion and fat transplantation studies [69]. These experiments demonstrated the crucial involvement of adipsin, a factor unique to adipose tissue, in the pathogenesis of arthritis. They concluded that adipose tissue plays a pivotal role in RA development, with adipsin being the important link between adipose tissue and arthritis [69].

The therapeutic application of BAT in the treatment of autoimmune diseases may have prospective benefits. Moon et al. [70] compared the BAT functions of mice with collagen-induced arthritis (CIA) to those of healthy mice. They demonstrated that CIA mice which had received BAT from healthy mice exhibited substantially reduced bone damage, inflammation, and cartilage damage. Additionally, the levels of pro-inflammatory cytokines, such as IL-6, IL-12, IL-17, and TNFα, were reduced in mice which had received BAT from healthy mice. According to these findings, the transplantation of normal BAT may have therapeutic significance in RA patients [70].

In a murine CIA model of RA, pathological changes were observed in the thoracic perivascular adipose tissue (PVAT) [71]. In humans, PVAT is the outermost layer of blood vessels surrounding most conduit vessels. In a healthy state, PVAT primarily releases anti-inflammatory molecules such as adiponectin, omentin, IL-10, nitric oxide (NO), and fibroblast growth factor-21 (FGF21), contributing to vascular homeostasis [72]. However, in pathological conditions such as obesity, PVAT undergoes significant changes, becoming predominantly composed of white adipocytes, and this results in the release of pro-inflammatory adipokines such as leptin, visfatin, chemerin, resistin, apelin, TNFα, monocyte chemoattractant protein-1 (MCP-1 or CCL2), IL-1β, IL-6, and IL-8 [72]. These pathological alterations in PVAT have been implicated in the development of CVD in RA patients [72].

However, not all observations support the link between high BMI and the development of RA [73,74]. Some data unanimously show a surprisingly protective action of obesity for radiographic joint damage in RA [75]. Possible explanations for this phenomenon include stimulation of bone synthesis due to increased mechanical loading, greater levels of oestrogens in obese patients known to exhibit bone-protective effects, as well as the involvement of adiponectin [75]. Another reason for this discrepancy may be that RA is associated with considerable alterations in body composition [76,77]. Body mass index is a widely utilized indicator of obesity; however, it is not perfect because it does not precisely reflect body fat distribution. A person with a normal BMI may have a high percentage of VAT, which is linked to an increased risk of cardiometabolic diseases [78]. In addition, approximately 30% of obese individuals have a favourable metabolic profile, meaning they do not have the metabolic complications typically associated with obesity [78]. The limitations of using BMI emphasise the need for improved methods of assessing body fat content. Newer methodologies, such as body composition analysis, can provide more exact measurements of body fat and its distribution [78].

Rheumatoid arthritis patients can have a condition known as sarcopenic obesity (SO), wherein they have a lower skeletal muscle mass and higher body fat mass when compared to healthy individuals [77]. The primary factor driving these changes is systemic inflammation; however, several other factors, including malnutrition, physical disability, and comorbidities, can also contribute to alterations in body composition in RA patients [77].

Treatment with corticosteroids and bDMARDs can influence body composition in RA patients, including inhibition of the inflammatory process and causing an increase in BMI. In particular, TNFα inhibitors have been shown to increase body weight and BMI as a potential side effect. A systematic review and meta-analysis found evidence for a small increase in body weight and BMI during treatment with TNFα inhibitors [79].

A comprehensive literature review by Letarouilly et al. [77] confirmed that RA is associated with a reduction in lean muscle mass and an increase in adiposity, regardless of the patient’s sex. Additionally, the prevalence of abnormal body composition conditions, such as excessive fat accumulation, sarcopenia, SO, and rheumatoid cachexia, is significantly greater among RA patients than among healthy individuals. Notably, these disturbances in body composition are observed before the initiation of DMARDs [77]. These findings highlight the importance of considering changes in body composition in the management and treatment of RA.

### 1.3. Significance of Adipokines in RA

**Figure 1:**
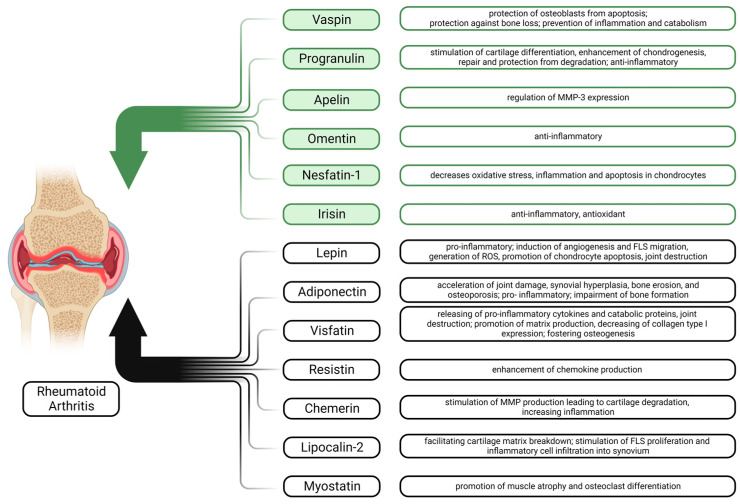
Adipokines and adipomyokines associated with rheumatoid arthritis (RA). This figure lists numerous adipokines and adipomyokines, along with their effects that may be related to RA. Created with BioRender.com (accessed on 15 October 2023).

An abnormal profile of adipokines in the blood indicates adipose tissue dysfunction in RA patients. Adipokines contribute to the pathogenesis of RA by influencing cartilage, synovium, bone, and numerous immune cells [80] (Figure 1).

Several adipokines, including leptin, adiponectin, and visfatin, have been shown to be elevated in patients with RA [81,82,83,84,85,86]. In the affected joints of RA patients, FLS, as well as osteoclasts, osteoblasts, and chondrocytes, produce several adipokines which contribute to the unique inflammatory microenvironment [80,84,86]. Due to alterations in systemic adipokine levels, their diagnostic potential as biomarkers has been suggested in the context of rheumatic diseases [87]. The significance of adipokines in RA lies in their potential to modulate the immune system and local cells in synovial tissue, cartilage, and bone [80,84,86].

A study by Giles et al. [88] found that the proportion of adipose tissue macrophages (ATMs), along with their characteristic crown-like structures, is elevated in the SAT of RA patients when compared to patients without RA. These alterations in ATMs were associated with the presence of autoantibodies, biomarkers of systemic inflammation, and insulin resistance (IR). In addition, the study [88] demonstrated that patients treated with DMARDs and TNFα inhibitors had lower ATM levels than other RA patients, which indicates that these medications might modulate the immune response and inflammation in adipose tissue of RA patients.

### 1.4. Adipose Tissue–Skeletal Muscle Cross-Talk in RA

The growing interest in changes in body composition associated with RA has highlighted the potential importance of cross-talk between adipose tissue and skeletal muscle in this disease [89]. Adipose tissue and skeletal muscle are two major organs that can actively communicate and interact with each other via the secretion of various factors, such as adipokines and myokines, which play crucial roles in modulating systemic inflammation, insulin sensitivity, and overall metabolic homeostasis [38,90,91]. Growing evidence shows that skeletal muscle abnormalities and adipose tissue dysfunction are common in RA patients [81,89,92]. These alterations to adipose tissue contribute to the chronic low-grade inflammation and metabolic disturbances seen in RA patients [81,93]. Simultaneously, skeletal muscle abnormalities such as wasting, impaired muscle function, and reduced exercise capacity are frequently observed in RA and contribute to diminished physical function and quality of life [89,94]. It is clear that RA is a systemic disease where chronic inflammation extends beyond the joints and affects multiple organ systems, including adipose tissue and skeletal muscle [89,95]. Cross-talk between adipose tissue and skeletal muscle is bidirectional, with adipose tissue-derived factors influencing muscle health and function and muscle-derived factors affecting adipose tissue metabolism and inflammation. In RA, dysregulation of this cross-talk may have deleterious effects on both adipose tissue and skeletal muscle, thereby exacerbating the disease process and comorbidities [81,89,90,93,96]. Understanding the complex relationship between adipose tissue and skeletal muscle in RA is crucial for the identification of novel therapeutic targets and interventions [77,97]. Targeting the factors implicated in fat–muscle cross-talk has the potential to reduce systemic inflammation, improve metabolic abnormalities, preserve muscle mass and function, and eventually enhance the overall management of RA [77,90,97,98].

Sarcopenia is defined by decreased skeletal muscle mass, strength, and function [89]. Although it is primarily associated with ageing, it can also occur in younger individuals with autoimmune disorders such as RA. These patients have a substantial risk of developing a condition known as rheumatoid sarcopenia, which is prevalent in over 25% of cases [77,97]. Chronic inflammation, driven by cytokines such as TNFα, IL-6, and IFN-γ, disrupts muscle homeostasis and accelerates muscle protein breakdown, hindering muscle stem cell renewal and impairing myofiber force [77,97]. This inflammatory burden sets RA apart from the more age-related variant, as seen in animal models [99] and RA patients [100], where skeletal muscle is a significant target of the inflammatory cascade.

Given its significant impact on mortality and disability, sarcopenia is of great clinical importance. Historically, RA research has centred on rheumatic cachexia, a condition marked by involuntary weight loss due to chronic illness. This state is characterised by diminished muscle strength, anorexia, fatigue, a low fat-free mass index, and abnormal blood parameters [89]. The traditional approach to investigating rheumatoid cachexia has primarily revolved around RA patients with lower body mass. However, contemporary studies have taken a new direction, exploring SO [101], which is characterised by reduced muscle mass and increased adipose tissue mass, particularly VAT. Notably, SO is more prevalent among individuals with RA than the general population, and its presence is linked to a less favourable prognosis [101]. In RA patients, a reduction in muscle mass may be accompanied by an increase in fat mass, which might result in the release of more pro-inflammatory molecules from VAT that could negatively affect skeletal muscles [89,101]. Approximately 12.6% of patients with RA are afflicted by SO [101].

Skeletal muscle fat infiltration occurs more rapidly in RA patients, adversely affecting muscle strength and physical performance [102]. Fat infiltration can affect skeletal muscle contractility and function, leading to metabolic dysfunction through lipotoxicity and insulin resistance [103]. Furthermore, intramuscular fat can release pro-inflammatory adipokines that induce myocyte apoptosis and contribute to systemic inflammation [104,105,106].

The use of glucocorticoids as a therapeutic strategy to mitigate manifestations of RA has been associated with the onset of sarcopenia [107]. Muscle proteolysis induced by glucocorticoids is predominantly facilitated by the activation of catabolic pathways, encompassing the ubiquitin–proteasome and autophagy–lysosomal systems. Furthermore, glucocorticoids induce muscle atrophy by altering the expression of pivotal regulatory factors involved in muscle development, such as insulin-like growth factor-I (IGF-I) and myostatin (MSTN) [108,109,110,111,112]. Myostatin, a robust suppressor of muscle hypertrophy, is upregulated by glucocorticoids, which subsequently instigate the phosphorylation of Smad2/3 and inhibit Akt phosphorylation, culminating in muscle atrophy [111]. These complex pathways are governed by specific transcription factors such as forkhead box O (FoxO), which are phosphorylated and inactivated by Akt in the cytoplasm [111].

Investigations into the correlation between glucocorticoid administration and sarcopenia in RA have yielded inconsistent results. A meta-analysis by Dao et al. [113] found that in adults with RA, glucocorticoid use was positively associated with sarcopenia. However, a recent meta-analysis by Tam et al. [114] did not identify a statistically significant relationship between baseline glucocorticoid administration and sarcopenia. Nevertheless, six out of seven studies reported elevated rates of glucocorticoid administration among RA patients with sarcopenia [8,9,10,11,12,13], with three studies indicating a significant difference [8,9,12]. While some studies have reported greater glucocorticoid doses in patients with sarcopenia compared to those without it [115,116], a study conducted by Brance et al. [117] did not identify an association between the cumulative dose of glucocorticoids and sarcopenia in univariate analysis.

A recent analysis of data from the CHIKARA study supports the hypothesis that glucocorticoids play a role in the development of rheumatoid sarcopenia [118]. Yamada et al. discovered that RA patients using glucocorticoids at an average dose of 3.25 mg/day or more for a year exhibited an increased risk of developing sarcopenia [118]. This observation is consistent with findings from an animal study conducted on TNF-α transgenic mice (TNF-tg). This study noted that muscle wasting was significantly augmented in animals that received glucocorticoids [119].

The conflicting findings on the relationship between glucocorticoid use and sarcopenia in RA patients underscore the need for further research in this area.

In their meta-analysis, Tam et al. [114] identified several studies that established a correlation between sarcopenia and increased disease activity in RA patients [120,121,122,123]. Upon pooled analysis, this association was found to include greater DAS28 scores among patients with sarcopenia [114]. The authors suggested that this correlation likely indicates a bidirectional relationship, as heightened disease activity may trigger the onset of sarcopenia through disability and inflammatory pathways [114,124]. The preliminary results of a longitudinal study by Park and colleagues [125] suggest that sarcopenia may be predictive of heightened disease activity in RA patients over a period of 3 years.

Sarcopenic obesity has been associated with poorer health outcomes when compared to sarcopenia or obesity alone. Previous studies have linked SO to an increased risk of cardiovascular disease, decreased bone mineral density, and heightened all-cause mortality [126,127,128]. However, few clinical studies have directly compared health outcomes between RA patients with SO versus those with sarcopenia or obesity alone. In a recent study, Baker et al. [101] investigated the association between sarcopenia, obesity, and SO and physical function and disability progression over time in RA patients. They found that RA patients with SO showed greater rates of physical disability and more severe progression of disability over time when compared to RA patients with sarcopenia or obesity alone.

Considering the multifaceted factors contributing to the development of RA, the implementation of a comprehensive therapeutic approach holds promise for enhancing overall health and bolstering muscle strength and function. A combined strategy that promotes physical activity and concurrently reduces systemic inflammation may offer synergistic benefits (for detailed review, see Bennett et al. 2023) [89].

No approved pharmacological treatment for sarcopenia is available, but various compounds have been investigated for their potential benefits to muscle health [129]. Several studies have indicated that antagonization of MSTN, activin A, and GDF11 signalling is a promising therapeutic approach for sarcopenia, and several smaller clinical trials have demonstrated that inhibition of MSTN/ActRII signalling may help to improve muscle mass in patients with muscle wasting [130]. Unfortunately, their effectiveness in RA patients with sarcopenia is still unknown. There has been increasing interest into how DMARDs affect muscle health in RA. While some studies have found these therapies to be effective, several recent systematic reviews and meta-analyses included studies of bDMARDs and reported no overall effect of these therapies on muscle mass [113,131,132,133].

The observations outlined in this review underscore the potential of interventions aimed at optimizing the endocrine profile of adipose tissue for practical clinical applications. Physical exercise is crucial in the prevention of sarcopenia and sarcopenic obesity [134,135,136,137,138]. The complexity of these health benefits can be partially explained by the release of bioactive substances into the bloodstream during exercise [138,139]. Resistance training promotes satellite cell activation and muscle protein synthesis and is crucial in combating sarcopenia. It enhances muscle mass and function by increasing muscle fibre size [140]. Systematic resistance exercises lead to an increase in the size of muscle fibres, particularly fast-twitch fibres [141], while aerobic exercise improves cardiovascular health, muscle oxidative capacity, and body mass control and may help to preserve adipose tissue mass while mitigating obesity [103,142,143,144]. Whole-body vibration, blood flow restriction (BFR), and neuromuscular electrical stimulation (NMES) are potential alternative treatments for sarcopenia, warranting further research [145,146,147].

In 2018, the European League Against Rheumatism (EULAR) established safe and practical physical activity guidelines for individuals with inflammatory arthritis [148]. Resistance training has been shown to improve the inflammatory state in RA patients, and a systematic review reported significant improvements in muscle strength [149]. However, the impact of various load masses and exercise modalities on muscle strength remains unclear due to limited studies. While excessive training could exacerbate joint destruction in RA patients [150], most studies suggest that moderate to high-intensity resistance training can be safely performed without worsening pain or disease activity [149,151]. Recent studies have demonstrated the muscle-strengthening effects of moderate to high-intensity training and suggest that a combination of resistance and aerobic exercises effectively improves muscle strength and could reduce total and visceral fat in RA patients [149,152,153,154,155,156,157,158]. In a study by Rodrigues et al. [159], low-load resistance training with BFR was found to effectively improve muscle strength in RA patients. Piva et al. [160] compared the effectiveness of NMES and high-intensity volitional resistance training in improving muscle structure and physical function in RA patients. Both methods were found to be effective, with NMES suggested as a viable alternative for patients who may not tolerate high-intensity resistance exercise.

A recent meta-analysis focusing on sarcopenia treatment in RA patients confirmed that exercise therapy enhances muscle mass in these individuals. Moreover, RA patients have shown positive responses and good tolerance to both resistance and non-resistance exercise therapies [161]. Alterations in the secretory profile of adipokines and myokines could partially explain the effect of exercise on muscle and adipose tissue. A recent study discovered that 12 weeks of resistance exercise reduced serum leptin levels commensurate with body fat mass or visceral fat area [157]. A recent animal study found that the increased expressions of certain genes in muscle atrophy (MSTN, atrogin-1, MyoD, and myogenin) in mice with experimental arthritis are restored back to control levels following acute resistance exercise [162].

Moreover, nutrition plays a significant role in influencing the progression and outcomes of inflammatory diseases such as RA. While research in this area is limited, some studies have linked dietary factors, including fatty acids, probiotics, and anti-inflammatory diets, to improved RA symptoms and daily functioning [163]. The impact of nutritional interventions on rheumatoid sarcopenia is discussed in detail in a recent review by Cruz-Jentoft et al. [164].

A comprehensive approach combining physical activity promotion, systemic inflammation reduction, nutritional interventions, and alternative treatments could potentially offer an effective strategy for managing rheumatoid sarcopenia. However, more research is needed to fully understand the impact of these interventions.

This review will assess the contribution of adipokines to the onset and progress of RA, with particular emphasis on the most extensively researched molecules in this field. Specifically, we will assess the current understanding of the role of adipokines in the pathogenesis of RA and their mechanisms of action in promoting inflammation and joint destruction. In addition, we will evaluate the potential of adipokines and adipomyokines as biomarkers for RA diagnosis, disease activity, prognosis, and the correlation between their serum and synovial fluid (SF) concentrations and disease severity and joint damage.

Furthermore, we will investigate the therapeutic potential of targeting specific adipokines and adipomyokines in the management of RA, focusing on strategies helping to reduce muscle wasting, improve metabolic regulation, and modulate the inflammatory milieu. Moreover, we will identify the challenges and opportunities associated with translating adipokine-based therapies into clinical practice, taking into account patient heterogeneity and developing targeted delivery techniques for specific tissues. By addressing these research objectives, this review aims to shed light on the complex interplay between adipokines, adipomyokines, and RA pathogenesis, highlighting their potential as biomarkers and therapeutic targets. Ultimately, this review aims to contribute to the development of personalised and efficient management strategies for RA patients.

## 2. Adipokines in Rheumatoid Arthritis

**Figure 2:**
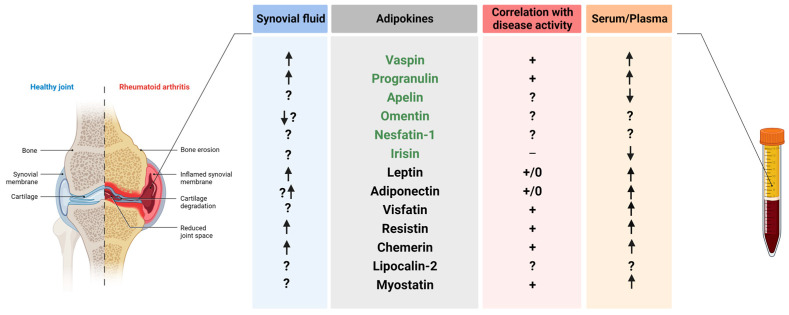
Levels of adipokines and adipomyokines in serum/plasma and synovial fluid in rheumatoid arthritis (RA), and their correlation with disease activity. This figure illustrates the changes in serum/plasma and synovial fluid levels of various adipokines and adipomyokines in RA, represented by upward (elevated levels) and downward (reduced levels) arrows. The figure also indicates the correlation of these substances with disease activity, denoted by a plus sign (positive correlation), minus sign (negative correlation), or zero (no correlation). A question mark is used to indicate conflicting or uncertain data. This comprehensive diagram aims to elucidate the complex relationships between these bioactive molecules, their systemic and local levels, and RA disease activity. Created with BioRender.com (accessed on 17 October 2023).

In rheumatic diseases, adipose tissue is not the sole source of adipokines. Other cell types, including chondrocytes and synoviocytes, also produce these mediators [87]. In musculoskeletal disorders, adipokines target cells and tissues such as bones, cartilage, and the synovial membrane via autocrine, paracrine, and endocrine pathways and play important roles in the pathogenesis of the disease [86,87]. Studies have reported elevated levels of adipokines in both serum and SF from RA patients when compared to healthy controls [165,166,167] (Figure 2).

However, Presle et al. [168] demonstrated that serum levels of adipokines do not serve as predictive markers for determining the concentration of adipokines in the SF. Moreover, their findings indicate that levels of adipokines within the synovial joint are regulated independently of the serum [168].

In recent years, there has been increasing interest in exploring adipokines as potential therapeutic targets and biomarkers in RA [80,169].

### 2.1. Leptin

Leptin was identified as the first adipokine, and it is predominantly secreted by WAT, with fluctuating levels throughout the day. The blood concentration of this hormone is proportional to the amount of adipose tissue in the body [170]. Leptin regulates energy balance and food intake by binding to functional receptors encoded by the diabetes (db) gene [170,171,172]. Leptin receptors (LepR) are class 1 cytokine receptors and are expressed by most of the immune cells. Signal transducers, such as Janus kinases (JAK), signal transducers and transcription activators (STAT), phosphatidylinositol 3-kinase (PI3K), and mitogen-activated protein kinase (MAPK), are activated when leptin binds to LepR [170,172]. Leptin has pleiotropic effects, influencing both adaptive and innate immunity [170,171].

Leptin plays a substantial role in the aetiology of RA, according to studies primarily conducted in mouse and rat models. Leptin was overexpressed in the SF of rats with experimental antigen-induced arthritis (AIA) [173]. In a study by Otvos et al. [174], the administration of leptin alone did not elicit arthritis in rats; however, it did exacerbate the clinical condition of mice subjected to K/BxN serum transfer arthritis. When rats in the same study received leptin receptor antagonists, leptin-induced disease activity was attenuated [174]. Another study in a murine model of CIA showed a significant increase in leptin levels in both joint tissue and SF compared to the control group. Upon injecting leptin into the knee joint of collagen-immunised mice, the onset of arthritis accelerated significantly, resulting in exacerbation of clinical symptoms and a notable increase in synovial hyperplasia, joint degeneration, and abundance of Th17 cells in the joint tissue [175].

Research conducted by Busso et al. [176] indicated that leptin-deficient (ob/ob) mice with AIA had lower levels of synovial inflammation and production of pro-inflammatory cytokines when compared to the control group. These findings suggest that leptin signalling contributes to the augmentation of synovial inflammation. On the other hand, in the proliferative arthritis model of zymosan-induced arthritis (ZIA), leptin appears to have a different function, as histopathology showed that ob/ob mice and mice with leptin receptor deficiency (db/db) had delayed arthritis resolution and more joint damage than controls [177]. High-fat diet (HFD)-induced obese mice which had CIA developed peripheral leptin resistance, reducing the severity and inflammation [178].

**Figure 3:**
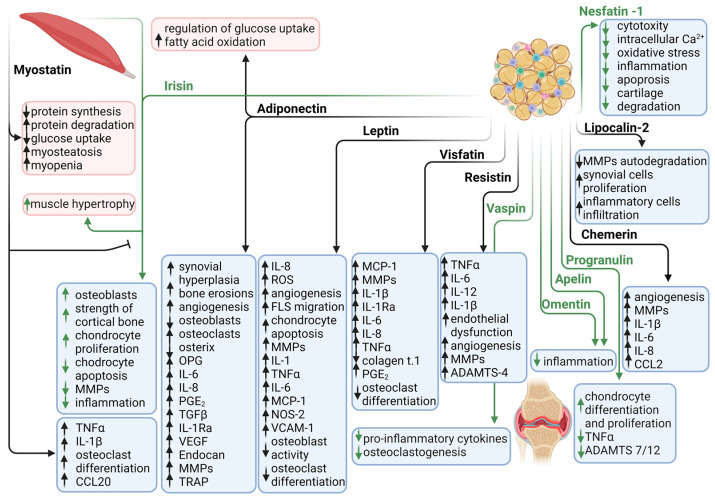
Schematic illustration of the role of adipokines and adipomyokines in the pathogenesis of rheumatoid arthritis. TNFα: Tumour Necrosis Factor alpha; IL-1β: Interleukin-1 beta; IL-6: Interleukin-6; IL-8: Interleukin-8; IL-10: Interleukin-10; IL-12: Interleukin-12; IL-1Ra: Interleukin-1 Receptor Antagonist; TGF Beta: Transforming growth factor beta; OPG: Osteoprotegerin; CCL2: C-C motif chemokine ligand 2; CCL20: Chemokine (C-C motif) Ligand 20; MCP-1: Monocyte Chemoattractant Protein-1; ADAMTS-4: A Disintegrin and Metalloproteinase with Thrombospondin Motifs 4; ADAMTS-7/12: A Disintegrin and Metalloproteinase with Thrombospondin Motifs 7/12; MMPs: Matrix Metalloproteinases; TRAP: Tartrate-Resistant Acid Phosphatase; NOS-2: Nitric Oxide Synthase 2; PGE2: Prostaglandin E2; ROS: Reactive Oxygen Species; VCAM-1: Vascular Cell Adhesion Molecule-1; VEGF: Vascular Endothelial Growth Factor; FLS: Fibroblast-like synoviocytes. Created with BioRender.com (accessed on 15 October 2023).

Identification of leptin receptors in FLS is consistent with the hypothesis that leptin plays a significant role in the pathogenesis of RA [179]. Specifically, leptin treatment led to increased IL-8 production in FLS, further indicating its pro-inflammatory effects in RA. The signalling pathways involved in this process include JAK2/STAT3, IRS-1/PI3K, Akt, and NF-κB, as well as the recruitment of p300, which is known to promote inflammation and may play an important role in the pathogenesis of RA [179]. Fibroblast-like synoviocytes migrate to unaffected joints, which contributes to the spread of RA [180]. Interestingly, leptin can induce the migration of FLS and angiogenesis by generating reactive oxygen species (ROS) [181] (Figure 3). Moreover, TNFα, IL-6, and IL-1β antagonists have been shown to attenuate leptin-induced ROS generation and FLS migration [181].

Expression of the LEPRb receptor in human chondrocytes provides compelling evidence of leptin’s influence on chondrocyte function [182,183]. Interestingly, the administration of exogenous leptin in rat knee joints elicited phosphorylation of STAT1 and STAT5 in chondrocytes, accompanied by increased proliferation and proteoglycan secretion [184]. Such observations suggested that elevated leptin levels may confer short-term protection against cartilage degradation [184,185]. However, prolonged exposure of human chondrocytes to leptin, as typically seen in obesity, has been associated with diminished cell viability [183,186,187]. In particular, up-regulation of LEPRb in leptin-treated human chondrocytes triggers mTOR activation, leading to altered cell proliferation and induction of cell senescence [188]. It was suggested that sustained activation of the leptin pathway in chondrocytes could contribute to cartilage degradation, while transient and low-level activation may exert a protective effect [183].

Leptin plays a role in the induction of chondrocyte apoptosis through its upregulation of LOXL3, activation of the mTOR pathway, and inhibition of autophagy [189,190]. Furthermore, leptin stimulates the production of MMPs and pro-inflammatory mediators such as IL-1, TNFα, IL-6, IL-8, and MCP-1 by chondrocytes. It also promotes the expression of adhesion molecules, including VCAM-1, contributing to cartilage degradation [191]. Moreover, leptin can contribute to the inflammatory process by synergistically acting with IL-1 to induce joint inflammation through the release of NOS type II (NOS2) from chondrocytes [192].

Leptin exerts its effects through local and central pathways in the regulation of bone homeostasis. Notably, Ducy et al. [193] conducted an experiment with ob/ob or db/db mice and hypothesised that leptin acts as a potent inhibitor of bone formation through the central nervous system. These observations have been validated by subsequent research [194,195]. Pogoda et al. [196] found that intracerebroventricular (ICV) administration of leptin in sheep led to significantly decreased osteoblast activity and trabecular bone mass. On the other hand, leptin inhibits osteoclast differentiation in peripheral blood mononuclear cells (PBMCs) and murine spleen cells cultured on bone substrates. This inhibiting effect is predominately mediated by the RANKL/RANK/OPG pathway, thereby substantially contributing to the suppression of bone resorption [197]

Elevated serum leptin in RA patients, when compared to healthy individuals, has been well established in numerous studies [85,167,198,199,200,201,202,203,204,205,206] (Table 1) and subsequently supported by meta-analyses [83,207]. Furthermore, leptin levels in the SF of RA patients were greater than in healthy controls [165,203] and individuals with osteoarthritis (OA) [198,199]. Leptin levels in SF are believed to result from the diffusion of adipokine from the circulation into the synovial tissue. However, recent studies in human chondrocytes have shown that these cells can produce leptin and express its long receptor, suggesting that this molecule may have a local function [208].

While some studies have reported a correlation between circulating leptin levels and disease activity markers such as C-reactive protein (CRP) and DAS28 [82,205,209,213,218,223,226,227,228,229,230,231,232,233], not all investigations support these associations [220,222,224]. In their meta-analysis, Lee et al. [82] concluded that a modest yet statistically significant correlation exists between leptin levels and disease activity parameters such as DAS28 and CRP. However, contrasting findings were reported by Hizmetli et al. [211], who found no statistically significant differences in plasma and SF leptin levels between RA patients and healthy controls. Additionally, they observed no correlation between leptin levels and disease duration, erythrocyte sedimentation rate (ESR), CRP, or erosive/non-erosive RA. In addition, Popa et al. [210] found an inverse correlation between plasma leptin concentrations and inflammatory markers in RA patients, indicating that chronic inflammation can inhibit leptin production (Table 1).

Recently, the possible involvement of leptin in the pathogenesis of joint erosions in RA has generated significant interest. Numerous research studies have elucidated this issue, revealing notable differences in leptin levels among patients diagnosed with erosive RA compared to those with non-erosive RA. The individuals with RA had notably elevated leptin levels in SF, although no significant difference was identified in plasma concentrations [198,199,217]. In addition, Olama et al. [203] found that the SF/serum ratio was significantly greater in RA patients with radiologic erosions, further supporting this observation. Based on these findings, the authors hypothesised that this decrease in serum leptin level could be due to local leptin uptake, supporting the hypothesis that leptin may play a protective role in the joint erosive process [203].

The multi-biomarker disease activity (MBDA) score, which includes 12 serum proteins, strongly predicts radiographic deterioration in RA. Leptin-adjusted MBDA scores were investigated in a recent study [234], and it was demonstrated that they were associated with clinical disease activity and predicted radiographic progression of RA better than the original score and other markers of disease activity [234,235]. Leptin antagonists have been proposed as potential preventative treatments for RA, offering a new avenue for personalised management of individuals at risk for developing RA, aiming to dampen the inflammatory cascade and mitigate the onset and impact of this disease [236,237]. Further research and clinical trials are needed to assess the safety and efficacy of these agents as a preventative approach.

The role of leptin in RA is not only associated with articular tissues; it might also have a potent effect on cell-mediated immune function (for review, see Wang et al., 2021, Tsuchiya and Fujio, 2022) [191,238].

### 2.2. Adiponectin

Adiponectin is a secretory protein with a molecular weight of 28–30 kDa; it is primarily produced by white adipocytes, encoded by the *ADIPOQ* gene and has multiple biological functions [239,240]. Adiponectin has also been found in various cell types, including osteoblasts, liver parenchyma cells, myocytes, endothelial cells, and the placenta [239]. It exists in three different isoforms based on its oligomerisation: low molecular weight (LMW), medium molecular weight (MMW), and high molecular weight (HMW). The MMW hexamers and HMW multimers are the most common forms found in the bloodstream, while monomers and LMW isoforms are present at low levels or not detected in circulation. Proteolytic cleavage of fibrous adiponectin produces globular adiponectin (gAPN), which may have its own biological activities. The HMW isoform of adiponectin is considered the most important physiologically and is increasingly used as a marker of adipocyte dysfunction related to pathological states [239,240,241]. Adiponectin binds to two main receptors, AdipoR1 and AdipoR2, which are distributed differently in various tissues. AdipoR1 activates AMP kinase, while AdipoR2 activates peroxisome proliferator-activated receptor alpha (PPARα), promoting fatty acid oxidation and glucose metabolism. Adiponectin has several biological functions, including stimulating fatty acid biosynthesis, inhibiting gluconeogenesis in the liver, and potentially affecting glucose uptake in skeletal muscles through signalling pathways. It improves insulin resistance by promoting fatty acid oxidation through the activation of PPARα and enhancing IRS signalling in skeletal muscle and liver. Additionally, adiponectin has anti-inflammatory and anti-atherosclerotic effects [239,240,241].

In contrast to leptin, individuals with obesity, type 2 diabetes (T2D), and metabolic syndrome have decreased adiponectin levels. The anti-inflammatory properties of adiponectin contribute to its beneficial effects on cardiovascular and metabolic disorders such as atherosclerosis and insulin resistance [242]. On the other hand, pro-inflammatory effects of adiponectin have been observed in conditions such as rheumatoid arthritis. However, the precise measurement of adiponectin isoforms and the lack of a universal standard have posed challenges in understanding these phenomena [239,240,241].

Adiponectin exhibits a multifaceted involvement in RA. In a CIA mouse model, inhibiting adiponectin led to reduced joint swelling and bone destruction and decreased expression of angiogenic markers [243]. Adiponectin injection in CIA mice resulted in earlier arthritis onset, accelerated joint damage progression, severe synovial hyperplasia, bone erosion, and osteoporosis. This was due to adiponectin-dependent Th17 cell response enhancement and upregulation of the RANKL/OPG ratio [244]. Adiponectin has been shown to promote inflammation in RA by stimulating the production of pro-inflammatory factors such as IL-6, IL-8, and prostaglandin E_2_ (PGE_2_) [245]. When exposed to adiponectin, FLS derived from RA patients produce increased levels of the above-mentioned factors [245].

Skalska et al. [246] examined how adiponectin and leptin affect the immune-modulating function of adipose mesenchymal stem cells (ASCs) from the infrapatellar fat pads of RA patients. Adipose mesenchymal stem cells were exposed to leptin, LMW, and HMW/MMW adiponectin isoforms. Unlike LMW adiponectin and leptin, HMW/MMW adiponectin significantly boosted the secretion of transforming growth factor β (TGF-β), IL-6, interleukin 1 receptor antagonist (IL-1Ra), PGE_2_, IL-8, and VEGF [246].

Adiponectin has also been linked to the dysregulation of joint tissue remodelling processes in RA. Adiponectin is involved in neovascularisation, a hallmark of RA, and induces the expression of VEGF in FLS and osteoblasts [247]. It also stimulates the expression of endocan, an endothelial dysfunction biochemical marker secreted by vascular endothelial cells. Endocan levels are increased in RA synovial tissues, and adiponectin stimulates its expression in RA FLS [247,248]. Adiponectin-stimulated FLS of RA patients have increased levels of VEGF and MMPs [249,250], which play crucial roles in angiogenesis, ECM degradation, and tissue remodelling. This upregulation by adiponectin suggests a potential mechanism by which it contributes to the destructive processes in RA joints [73]. Huang et al. [243] conducted a study to investigate the role of adiponectin in angiogenesis in RA. They found that adiponectin increases the expression of VEGF in a dose- and time-dependent manner, which stimulates the formation and migration of endothelial progenitor cells (EPCs). These angiogenic activities induced by adiponectin were facilitated by MEK/ERK signalling. In vivo experiments confirmed that adiponectin downregulates microRNA-106a-5p (miR-106a-5p) [243].

Furthermore, adiponectin impairs osteoblast mineralisation capacity and enhances osteoclast bone-resorptive activity [251]. It promotes the expression of MMP-9 and tartrate-resistant acid phosphatase (TRAP) while increasing IL-8 secretion in osteoblasts. Additionally, adiponectin inhibits osterix expression in RA-induced human bone tissue and induces osteoprotegerin mRNA expression, leading to impaired bone formation [251]. Qian et al. [252] demonstrated that adiponectin promotes osteopontin production, which recruits osteoclasts to the bone surface and initiates bone erosion.

These observations diverge from previous findings in healthy cells [253,254], suggesting that the chronic inflammatory environment in RA alters the typical physiological response to adiponectin [255], shifting from anti-osteoclastogenic to pathologically pro-resorptive. As a result, adiponectin contributes to bone damage in RA by directly inhibiting osteoblast differentiation and promoting osteoclastogenesis, leading to increased bone resorption [255].

Specific antibodies against adiponectin isoforms were designed to specifically bind to MMW and HMW adiponectin. This targeted approach resulted in a reduction of IL-6 and IL-8 induction in osteoblasts that had been stimulated by these isoforms [256]. Furthermore, Lee et al. demonstrated that antibodies targeting both MMW/HMW and MMW isoforms significantly improved CIA in mice, suggesting that both adiponectin isoforms may contribute to the progression of RA [256].

Another study [257] revealed that while T follicular helper cells (Tfh) did not directly respond to adiponectin, adiponectin indirectly affected these cells by activating them through FLS stimulation, mediated primarily by IL-6. In support of these findings, Liu et al. demonstrated that intra-articular administration of adiponectin led to increased synovial inflammation and a higher frequency of Tfh cells in mouse CIA [257].

Numerous studies have demonstrated elevated adiponectin levels in the serum and SF of RA patients when compared with healthy controls or individuals with OA [200,202,219,258,259,260,261,262,263,264,265,266,267,268] (Table 2). A meta-analysis encompassing 11 studies, including a cohort of 813 RA patients and 669 healthy controls, showed a significant elevation in circulating adiponectin levels among RA patients compared to the control groups [83]. A 29-year follow-up study of subjects with obesity found that those with high serum adiponectin levels at baseline were more likely to develop RA [269]. Furthermore, Tan et al. [270] conducted a study that revealed abundant expression of adiponectin and its receptors, namely AdipoR1 and AdipoR2, in the synovial membrane of RA patients, particularly within FLS.

Several investigations [200,260,261,262,265,272,275,277,278] have found a positive correlation between adiponectin serum levels and disease activity or radiographic progression. Nevertheless, this association is not universally acknowledged, as other studies have produced contradictory findings [202,219,223,258,268,279]. Baker et al. [276] recently found that adiponectin levels were strongly associated with radiographic damage, seropositivity, longer disease duration, prednisone use, and circulating inflammatory cytokines in people with rheumatoid arthritis. Nevertheless, they did not find a significant association between adiponectin levels and DAS28 (Table 2). However, it has been postulated that adiponectin holds promise as a valuable therapeutic biomarker to monitor the severity, progression, and treatment outcomes of RA patients [261].

### 2.3. Visfatin

Visfatin is a multifunctional adipokine, also known as nicotinamide phosphoribosyltransferase (NAMPT). It is predominantly synthesised in large quantities in visceral adipose tissue but is also expressed in numerous other organs and tissues, including the bone marrow, liver, musculature, heart, placenta, lungs, and kidneys [280,281]. Its role in inflammation and immune modulation has generated considerable interest. Visfatin, initially identified as an immune-modulating cytokine, has been shown to influence the production of B cells, which are involved in the humoral immune response, and activate T cells, which play a crucial role in the cell-mediated immune response [280,281,282]. Visfatin’s immune-modulating properties have been elucidated, shedding light on its function in stimulating the secretion of MCP-1, a chemokine that facilitates the recruitment of immune cells to inflamed tissues. In addition, visfatin increases the expression of MMPs, enzymes involved in tissue remodelling [283,284]. Remarkably, visfatin activates key inflammatory signalling pathways, such as nuclear factor kappa B (NF-κB), MAPK, and PI3K, thereby amplifying the inflammatory response [281,283,284]. When visfatin concentrations are increased, several pro-inflammatory mediators, including IL-1β, IL-1Ra, IL-6, IL-8, and TNFα, are expressed at elevated levels [280,281].

Studies in humans and experimental animal models suggest that visfatin may play a significant role in the development of RA [166,200,202,260,285,286,287,288,289,290,291]. In studies employing experimental animal models, elevated visfatin levels were associated with RA development [289,290,291]. Additionally, a pro-inflammatory cytokine, IL-6, augmented the increase in visfatin [289]. In a murine model of RA, visfatin expression in immune cells was linked to the production of IL-6, the infiltration of immune cells, the proliferation of Th17 cells, and the presence of autoantibodies associated with RA [292]. Experimental studies utilising visfatin blockers have shown promising results in attenuating pathological processes associated with RA [291,292]. Notably, visfatin deficiency slowed the progression of RA, as evidenced by reduced bone degradation and inflammation [292,293].

Visfatin expression has been detected in various cell types in the synovial tissue of RA patients, including adipocytes and endothelial cells, with the highest expression observed in FLS [289,294,295]. Visfatin can induce the activation of FLS, causing the release of pro-inflammatory cytokines and catabolic proteins, which can lead to joint destruction [294,295]. Furthermore, visfatin was found to stimulate its own expression in FLS [294]. Studies by Hasell et al. [296]. have demonstrated that visfatin plays a role in facilitating adhesion of FLS to the endothelium and contributes to the early stages of the disease process within the context of the adhesion cascade.

Visfatin is involved in matrix production during the differentiation of mesenchymal stem cells (MSCs) and decreases collagen type I expression, highlighting its significance in bone pathophysiology [297]. Gosset et al. [298] demonstrated that visfatin triggered the release of PGE_2_ and the expression of catabolic enzymes in human chondrocytes, indicating a potential role for visfatin in cartilage degradation. Visfatin fosters osteogenesis by enhancing runt-related transcription protein 2 (Runx2) expression. Inhibition of visfatin in mouse bone marrow-derived MSCs (BM-MSCs) reduces osteoblastogenesis and hinders osteoblast differentiation markers, alkaline phosphatase activity, and matrix mineralization [299]. It was demonstrated that visfatin could impede RANKL-induced osteoclast differentiation by disrupting survival-related signalling pathways [300].

It was demonstrated that visfatin levels are greater in RA patients than in healthy individuals [166,200,202,260,285,286,287,288]. A meta-analysis of 11 studies found that circulating visfatin levels were significantly greater in RA patients than in healthy controls [83]. Additionally, there was a positive correlation between visfatin levels and anti-CCP antibodies and the progression of RA [166,221,271,294]. Some studies have also found a positive correlation between visfatin levels and disease activity [166,221,260,294]. Rho et al. [202] demonstrated a positive association between visfatin levels and radiologically detectable joint damage in RA patients after adjusting for confounding factors (Table 3).

Additionally, PBMCs and peripheral blood granulocytes (PBGs) from RA patients had greater levels of visfatin expression when compared to healthy controls [286]. Visfatin levels were also greater in the synovium of RA patients when compared to healthy controls and OA patients [289]. Additionally, elevated visfatin levels in RA synovial joints positively correlated with inflammation and clinical disease activity, further supporting the role of visfatin in joint alterations associated with RA [294].

In their comprehensive review, Franco-Trepat et al. [303] provided compelling evidence which showed that visfatin plays a crucial role in RA inflammatory and catabolic processes. The authors proposed the existence of a positive feedback loop between visfatin expression and various inflammatory factors, potentially contributing to the persistence of inflammation in RA [303]. Several authors have acknowledged the association between visfatin and RA activity and progression, underscoring its potential as a promising therapeutic target [80,300,303,304,305,306]. Moreover, this association has sparked interest in exploring visfatin’s role as a biomarker and its potential as a predictor of response to biologic treatments [285,287,288,302,303,307].

### 2.4. Resistin

Resistin is a cysteine-rich peptide hormone encoded by the RETN gene, which belongs to a family of secreted proteins known as resistin-like molecules (RELMs) or found in inflammatory zone (FIZZ) proteins and is secreted by adipose tissue and other cells, such as mononuclear leukocytes and macrophages [308]. Resistin has been proposed to link obesity, insulin resistance, and diabetes in rodents, as it antagonises insulin action and impairs glucose homeostasis [169,308].

In addition to its metabolic effects, resistin has been implicated in various inflammatory and cardiovascular diseases. Resistin may contribute to the pathogenesis of these diseases by modulating the immune response and inducing various pro-inflammatory cytokines. Resistin may also affect vascular function by inhibiting endothelial nitric oxide synthase (eNOS) activity, which promotes endothelial dysfunction, thrombosis, angiogenesis and smooth muscle cell proliferation [308,309,310]. Moreover, resistin has been implicated in the pathogenesis of autoimmune inflammatory diseases, including RA. In recent years, researchers have explored the possible involvement of resistin in RA pathogenesis and its potential as a biomarker and therapeutic target.

The administration of resistin to human articular chondrocytes resulted in the upregulation of several cytokines and chemokines, including TNFα, IL-6, and IL-12 [311]. Additionally, resistin treatment increased the expression of various catabolic enzymes and markers associated with cartilage degradation, such as MMP-1, MMP-2, and ADAMTS-4 [311]. Bokarewa et al. [312] demonstrated that intra-articular injection of recombinant resistin in healthy mice induced a joint inflammation similar to human arthritis [312]. They also showed that in response to extracellular resistin, both human PBMC and synovial leukocytes produce various pro-inflammatory cytokines, such as TNFα, IL-1β and IL-6. Remarkably, resistin stimulates its own production in human PBMC, establishing a positive feedback loop.

Additionally, when exposed to TNFα but not IL-1β or IL-6, PBMCs demonstrate an induced expression of resistin. The study provided evidence indicating that resistin, expressed in the synovial tissue of individuals with RA, plays a role in the pathogenesis of the disease by enhancing FLS chemokine production [312,313].

In a recent study [314], researchers employed the AIA-mouse model to investigate the effects of intra-arterial resistin administration on PVAT function and showed that resistin administration led to PVAT dysfunction. These findings are particularly intriguing, considering that PVAT is known to primarily release pro-inflammatory adipokines under pathological conditions, including resistin [72]. These observations suggest that resistin might play a role in a pathological positive feedback loop, whereby PVAT dysfunction leads to increased resistin production, further impairing PVAT function. These findings have important implications for our understanding of how PVAT is involved in the development of CVD in RA.

Some studies have demonstrated that resistin levels are elevated in the serum and SF of RA patients when compared to healthy subjects and OA patients [165,200,202,206,216,312,315,316,317,318,319]. However, some studies [219] found no significant difference in serum resistin levels between RA patients and healthy individuals [219,260] (Table 4). The expression of resistin has been documented by Šenolt et al. [320] in various cell types present in the synovial tissue, including FLS, as well as in distinct inflammatory cell types observed in the synovium of RA patients, namely macrophages, B lymphocytes, and plasma cells [320]. Several researchers have documented positive correlations between serum resistin levels and markers of inflammation, such as ESR and CRP, as well as clinical disease activity as measured by DAS28 in RA patients [206,219,223,228,315,318,320].

Bokarewa et al. [312] found that the resistin levels in SF of RA patients were significantly greater than in those with OA or other primarily non-inflammatory joint diseases. However, they observed lower resistin concentrations in the serum of RA patients, compared with the matched SF samples, indicating a potential increase in local production or selective accumulation of this adipokine at the site of inflammation [312]. In a meta-analysis encompassing eight studies involving 620 patients with RA and 460 healthy controls, it was found that serum resistin levels in RA patients were significantly elevated when compared to those in the control group [323]. Anti-TNFα therapy reduced serum resistin levels in RA patients, indicating a strong correlation with inflammatory markers [324,325]. In the early phases of active RA, it has been proposed that measuring resistin concentration could be a valuable biomarker for identifying individuals at high risk of developing erosive disease [322].

### 2.5. Other Adipokines

#### 2.5.1. Vaspin

Vaspin is classified as an adipokine belonging to the serine protease inhibitor family. Its expression has been observed in both visceral and subcutaneous adipose tissue in adult humans [326]. Its regulation may vary depending on the specific adipose tissue depot and is associated with obesity, glucose metabolism, and insulin resistance. The expression of vaspin is notably high in the liver and skin, while it is moderately expressed in the brain, heart, and spleen. The mRNA expression of vaspin is elevated in individuals who suffer from obesity and T2D [326].

Furthermore, a positive correlation exists between circulating vaspin and vaspin mRNA expression levels in adipose tissue. Several studies have postulated that vaspin can enhance ATK signalling and inhibit NF-κB signalling in adipocytes, increasing insulin sensitivity in both adipocytes and hepatocytes [326,327]. Additionally, vaspin has been found to attenuate the pro-inflammatory cytokine response induced by IL-1β in adipocytes [326,327].

In in vitro studies, human osteoblasts were protected from apoptosis, which may be attributed to the activation of the MAPK/ERK signalling pathway [328]. In their research, Kamio and colleagues [329] noted that vaspin inhibited RANKL-induced osteoclastogenesis. Notably, vaspin demonstrated inhibitory effects on the expression of nuclear factor of activated T cells c1 (NFATc1) induced by RANKL. Moreover, it inhibited the enhancement of MMP-9 and cathepsin K induced by RANKL. The authors suggested that vaspin could downregulate osteoclastogenesis by partially suppressing the transcription factor NFATc1. A study by Wang et al. [330] revealed that vaspin has a protective effect against bone loss induced by an HFD.

Furthermore, it showed that vaspin facilitated the osteogenic differentiation process by activating the Smad2/3-Runx2 signalling pathway. This observation seems contradictory to previous findings by Liu et al. [331], who observed that vaspin inhibits osteogenic differentiation in the pre-osteoblast cell line MC3T3-E1 and proposed that this inhibition is mediated through a regulatory loop involving the PI3K-Akt signalling pathway and miR-34c. These contradictory results could be due to several factors, including differences in the experimental models used (pre-osteoblast cell line MC3T3-E1 vs. primary rat osteoblasts). Additionally, Bao et al. demonstrated that vaspin prevented leptin- and IL-1β- induced inflammation and catabolism by inhibiting the activation of NF-κB in rat chondrocytes [332,333].

The complete evaluation of vaspin’s potential role in rheumatic conditions such as RA is still incomplete. Multiple sources [225,262,334,335] indicate that individuals with RA have elevated serum vaspin levels when compared to healthy subjects (Table 5). Vaspin levels in SF were significantly greater in patients with RA than in OA patients [334]. Furthermore, a correlation was observed between vaspin levels and DAS28 in the RA cohort. However, the study found no correlation between serum vaspin levels and serum CRP levels or leukocyte count in the SF of RA patients [334].

Maijer et al. [351] have suggested that serum vaspin levels may be a potential biomarker for predicting the development of RA in autoantibody-positive individuals.

#### 2.5.2. Chemerin

Chemerin, encoded by the *Rarres2* gene, is a versatile protein with diverse functions in inflammation, adipogenesis, angiogenesis, and energy metabolism. As a small chemotactic protein, chemerin is secreted as an inactive prochemerin and requires proteolytic activation by serine proteases to unleash its biological activity. It binds to three G protein-coupled receptors: chemokine-like receptor 1 (CMKLR1/chemerin1), G protein-coupled receptor 1 (GPR1/chemerin2), and CC-motif chemokine receptor-like 2 (CCRL2), found on a variety of cells, including DCs, macrophages, and natural killer cells, where they regulate chemotaxis toward the site of inflammation and activation state. Predominantly expressed in adipocytes and immune cells, CMKLR1 is a key receptor for chemerin signalling [352,353]. In humans, chemerin levels positively correlate with BMI and obesity-related biomarkers. It is highly expressed in WAT, liver, and lung, which suggests its involvement in energy homeostasis and metabolic regulation. Chemerin acts through CMKLR1 to influence adipogenesis, angiogenesis, and inflammation within adipose tissue. It is implicated in metabolic disorders such as metabolic syndrome, insulin resistance, and obesity, acting as a pro-inflammatory adipokine with complex endocrine, paracrine, and autocrine effects. However, its role as a pro- or anti-inflammatory modulator remains unclear, as chemerin can exhibit anti-inflammatory properties under specific conditions [352,353].

Chemerin stimulates FLS to produce metalloproteinases, especially MMP-3, which results in cartilage degradation and joint degeneration [354]. Chemerin also exacerbates inflammation in patients with RA by inducing the production of various pro-inflammatory cytokines, such as IL-1β and IL-6 [354].

Additionally, greater chemerin concentrations cause increased MMP-2, MMP-3, MMP-13, and IL-8 production in RA patients [355]. In addition, chemerin-stimulated chondrocytes in RA patients can induce other molecules involved in cartilage degradation, such as C-C motif ligand 2 (CCL2) [356]. Chemerin also facilitates the migration of immune cells and FLS to the joints, accelerating cartilage degradation [354]. These results suggest that chemerin plays a role in joint inflammation and cartilage destruction. Elevated chemerin serum levels were observed in RA patients when compared to healthy controls [302,335]. Additionally, the chemerin concentration in SF of individuals with RA was significantly increased, which is primarily due to the robust chemerin production by FLS [354,356]. Chemerin is also closely associated with the severity and activity of RA, making it a useful biomarker [302,336,343] (Table 5). Furthermore, Vazquez-Villegas et al. [344] found a correlation between elevated chemerin levels and functional disability in RA patients.

#### 2.5.3. Omentin

Omentin-1 and omentin-2 are exclusively secreted by adipose tissue depots. In a study by Yang et al. [357], the gene expression of these molecules was discovered in visceral stromal vascular cells but not in adipocytes. Omentin-1 is the predominant isoform in human plasma, and its expression is predominantly observed in omental adipose tissue but not subcutaneous adipose tissue [358]. Omentin-1 has anti-inflammatory properties and plays crucial functions in regulating glucose homeostasis, lipid metabolism, insulin resistance, and the development of diabetes [359]. Omentin can enhance insulin signal transduction by activating Akt/PKB, affecting adipose tissue distribution [357].

There is currently limited knowledge regarding the role of omentin in RA. Nonetheless, some studies have suggested that omentin may be involved in the pathogenesis of RA. Maijer et al. [351] found a positive correlation between serum omentin levels and CRP in individuals with an increased risk of developing RA who tested positive for autoantibodies. Arias-de la Rosa et al. [206] observed that serum omentin levels in RA patients were significantly greater than in controls and a positive correlation between omentin serum levels and the DAS28. Robinson et al. [360] found a correlation between omentin and MMP-3 levels in patients with mild RA but not in those with severe RA. Wahba et al. [335] observed that omentin serum levels were lower in RA patients than in healthy controls. In addition, Senolt et al. [334] demonstrated that the SF levels of omentin in individuals with chronic-inflammatory RA were significantly lower than those in individuals with OA (Table 5).

#### 2.5.4. Progranulin

Human progranulin (PGRN) is a 75–80 kDa glycoprotein composed of seven granulin/epithelin repeats. It is biologically active, with anti- and pro-inflammatory effects. Originally described as an autocrine growth factor, PGRN stimulates chondrocyte differentiation and proliferation and has been identified as an adipokine with anti-inflammatory properties due to its competitive binding to TNFα receptors [361,362]. Several cells, including adipocytes, macrophages, and chondrocytes, secrete PGRN. Emerging evidence shows that PGRN is protective in immune-mediated diseases, including RA [363].

Human progranulin is a potent stimulator of cartilage differentiation [361]. It enhances cartilage chondrogenesis and repair by modulating BMP2 signalling and protects cartilage from degradation and bone resorption by activating the ERK1/2 and JunB pathways while inhibiting NF-κB and TNFR1 pathways. Additionally, PGRN suppresses TNFα and ADAMTS-7/12, which are involved in cartilage degeneration in arthritis [361,364]. Moreover, PGRN regulates miR-138, which targets histone deacetylase 4 and affects NF-κB levels in RA [365], attenuates the inhibitory effects of TNFα on osteoblast differentiation, and prevents cartilage oligomeric matrix protein (COMP) degradation [365]. Treatment with PGRN has been shown to prevent the loss of proteoglycans and to prevent the expression of inflammatory biomarkers in human cartilage [366]. Other studies have shown that PGRN can activate anabolic pathways and inhibit catabolic metabolism in chondrocytes by binding to TNFR2 and blocking TNFR1 and also negatively modulates Wnt/catenin signalling, reducing osteophyte formation and cartilage degeneration [367,368,369,370].

Recently, the potential role of PGRN as a biomarker and a therapeutic agent has been suggested [368]. The complete PGRN protein exhibits anti-inflammatory characteristics; however, it is not a viable therapeutic target due to its multifunctional nature in promoting tumourigenesis and its susceptibility to proteolytic cleavage, resulting in the formation of pro-inflammatory granulins [368]. In order to address these limitations, a novel protein known as Atsttrin was engineered [368,371].

Tang et al. [371] investigated the role of PGRN as a modulator of TNFα/TNFR signalling and its therapeutic potential for RA. They showed that PGRN acts as an endogenous, competitive TNFα antagonist by binding to TNFR and blocking its interaction with TNFα. They also demonstrated that PGRN deficiency exacerbates arthritis inflammation in a CIA model, while recombinant PGRN administration ameliorates it. Furthermore, they compared the anti-inflammatory effects of PGRN and Atsttrin, a synthetic protein derived from three PGRN fragments, which has enhanced TNFR affinity. They found that both proteins reduced arthritis severity in various models, but Atsttrin was more potent than PGRN in inhibiting inflammation [371].

Some studies have reported the clinical relevance of PGRN in RA. These studies consistently show that RA patients have greater levels of PGRN in their serum than healthy individuals, regardless of sex and age [337,338,339]. In RA patients, PGRN was associated with disease activity [338,339,340] (Table 5), while the ratio of PGRN to TNFα closely correlated with the progression of RA [337]. A study by Chen et al. [287] noted greater populations of human B regulatory cells (Breg) in RA patients; however, Breg cell numbers did not correlate with PGRN level, suggesting an independent alteration in RA [339]. Levels of PGRN are significantly greater in the SF of RA patients than in OA patients [337,339], with immunohistological analysis of synovial tissue from RA patients confirming this upregulation of PGRN in inflammatory cells [338].

#### 2.5.5. Lipocalin 2

Lipocalin-2 (LCN2), or neutrophil gelatinase-associated lipocalin (NGAL), is a glycoprotein from adipose tissue that modulates inflammation and metabolism and has been linked to obesity, hyperglycaemia, and insulin resistance [372]. In chondrocytes, LCN2 is produced in response to IL-1β, leptin, adiponectin and LPS [373,374]. In these cells, it binds to MMP-9 and prevents its auto-degradation [375,376], which may facilitate cartilage matrix breakdown, as MMP-9 degrades cartilage components [374,375]. Moreover, LCN2 stimulates synovial cell proliferation and inflammatory cell infiltration in RA synovium [376]. LCN2 has been proposed as a biomarker of cartilage degradation in arthritic diseases; however, additional investigations are required to validate this hypothesis [377].

#### 2.5.6. Nesfatin-1

Nesfatin-1 is an anorexigenic molecule that plays a crucial role in the regulation of energy homeostasis. It is secreted by the hypothalamus and other tissues, including SAT, stomach, pancreas, and testes [378]. The expression of nesfatin-1 has also been observed in chondrocytes of both human and mouse origin [379]. Xu et al. [380] evaluated the effects of nesfatin-1 on acidosis-stimulated chondrocyte injury in vitro and in vivo, focusing on the involvement of acid-sensing ion channel 1a (ASIC1a) and its mechanism of action in RA. In vitro experiments showed that nesfatin-1 decreased cytotoxicity and intracellular Ca^2+^ levels and attenuated oxidative stress, inflammation, and apoptosis in chondrocytes. In vivo, the analysis revealed that nesfatin-1 ameliorated cartilage degradation and decreased ASIC1a expression in chondrocytes of rats with RA.

Chang et al. [381] analysed gene expression in synovial tissue samples from RA patients and CIA mice. Their findings revealed higher levels of nesfatin-1 and osteoclast markers in these samples compared to those from normal synovium. Sequencing of RNA revealed that nesfatin-1 increased Bone morphogenetic protein 5 (BMP5) expression in FLS, while short hairpin RNA reduced BMP5 and osteoclast formation in CIA mice [381]. Patients with severe disease had greater serum nesfatin-1 levels, which positively correlated with greater CRP and ESR concentrations [341]. Nesfatin-1 levels in the synovium were significantly elevated in patients with RA when compared to the control group. Furthermore, a positive correlation was observed between nesfatin-1 levels in the synovium and the presence of RF in patients with RA [345] (Table 5).

#### 2.5.7. Apelin

Apelin, a protein found in numerous tissues, is a natural ligand for the apelin receptor (APJ) which has anti-inflammatory properties and inhibits the NF-κB and ERK1/2 signalling pathways. In cases of obesity, both adipose tissue and plasma apelin levels are elevated [169,382]. Evidence suggests that apelin might be involved in RA, as early-stage RA patients have lower levels of this peptide than healthy individuals [383]. More recently, Wahba et al. [335] demonstrated decreased apelin serum levels in RA patients (Table 5) and revealed a negative correlation between apelin and NF-κB levels. Furthermore, the same study revealed an inverse correlation between apelin levels and MMP-3 levels in RA patients, indicating that decreased apelin promotes MMP-3 expression via NF-κB induced transcription [335].

### 2.6. Adipomyokines

Numerous molecules are actively secreted by both skeletal muscle cells and adipocytes. These molecules, known as adipomyokines, play a pivotal role in metabolic pathways and are instrumental in facilitating muscle growth, regeneration, and intricate communication among various tissues such as muscles, liver, WAT, BAT, brain, and bone [384].

Irisin and MSTN are among the best-characterised adipomyokines, exhibiting significant interdependence in the context of adipose tissue muscle tissue crosstalk. This intricate relationship is of considerable significance and can potentially contribute to RA pathogenesis, with particular emphasis on SO.

#### 2.6.1. Myostatin

Myostatin, alternatively referred to as growth differentiation factor 8, is a myokine that has been thoroughly investigated due to its profound influence on muscle and adipose tissue [385,386,387]. It is predominantly expressed in skeletal muscle and also in WAT, BAT, and cardiac muscle [388,389,390]. Being a member of the transforming growth factor superfamily, MSTN exerts its effects through interaction with the ActRIIB receptor, leading to the phosphorylation of the Smad2 and Smad3 proteins [391]. This phenomenon results in the suppression of protein synthesis in skeletal muscle by inhibiting the IGF-1/Akt/mTOR pathway [391]. Consequently, the activation of genes implicated in muscle protein degradation is observed concomitantly with the inhibition of protein synthesis. Furthermore, MSTN plays a significant role in muscle atrophy via the FoxO1 signalling pathway while also exerting an inhibitory effect on glucose uptake in skeletal muscle by downregulating GLUT4 and AMPK activity [385,386,387,391].

In animal obesity models and obese humans, MSTN is upregulated [392,393]. Regular physical activity decreases MSTN expression in the skeletal muscles of obese individuals [393,394]. Studies have demonstrated a positive association between MSTN levels and intramuscular adipose tissue, suggesting a potential involvement of this myokine in the development of myosteatosis [103]. Follistatin is a protein which binds to MSTN and inhibits its function, promoting muscle hypertrophy. Exercise has been shown to increase circulating levels of follistatin [395].

The pivotal role of MSTN in the development of RA is widely acknowledged in the scientific literature [90,346,347,396,397,398,399], as it upregulates TNFα and IL-1β expression through the PI3K-Akt signalling pathway in FLS, promoting muscle atrophy and osteoclast differentiation [396]. Hu et al. [400] found that MSTN and IL-1β levels in synovial fluid from RA patients were overexpressed and positively correlated, and MSTN dose-dependently regulated IL-1β expression through the ERK, JNK, and AP-1 signal-transduction pathways. In a mouse model of RA, it has been demonstrated that MSTN acts via the myostatin-CCL20-CCR6 pathway to promote the migration of Th17 cells to inflamed joints. Interestingly, IL-17A strongly regulates the expression of MSTN in FLS [398]. The authors hypothesised that elevated MSTN levels contribute to the secretion of CCL20, which further facilitates the infiltration by Th17 lymphocytes. As a result, the interaction between activated FLS and Th17 cells, mediated by MSTN and IL-17A, establishes a negative inflammation feedback loop. This maintains the continuous infiltration by Th17 cells, thereby contributing to the persistence of chronic joint inflammation [398]. Expression of MSTN is elevated in the synovial tissues of RA patients and hTNFtg mice, an animal model of RA. Myostatin increases RANKL-induced osteoclastogenesis in vitro by regulating NFATC1 via SMAD2 [399]. Deficiency of MSTN or its neutralisation reduces the severity of arthritis in hTNFtg mice, primarily through a decrease in bone resorption. Likewise, in the K/BxN serum-transfer arthritis model in rodents, MSTN ablation increases grip strength and decreases bone erosion [399].

Greater plasma MSTN levels have been observed in RA patients when compared to healthy controls, along with their association with disease activity and inflammatory biomarkers [346,347,348] (Table 5). Elevated MSTN levels were shown to increase the risk of rheumatoid cachexia [346,348] in RA patients. Lin et al. [347] found that elevated levels of MSTN in the bloodstream were associated with cumulative joint injury in a cohort of RA patients. This finding provided compelling evidence for the intricate relationship between muscle and bone in the context of RA. Additionally, the researchers observed a synergistic interaction between elevated serum MSTN levels and baseline loss of skeletal muscle in RA patients [347], which could be used to predict the progression of radiographic joint injury over one year. These findings emphasise the probable involvement of MSTN and muscle–bone interactions in RA disease progression as prognostic factors [347].

Myostatin is a negative regulator of skeletal muscle mass growth and development [386,387] and has been suggested as a possible biomarker for decreased muscle mass in RA patients. However, data regarding the relationship between MSTN levels and muscle health in RA have been inconsistent [89,346,347,348,397,401]. This discrepancy may be due to the observed correlation between MSTN levels and RA disease activity [346]. Specifically, the increase in MSTN may be partially due to the inflammation inherent in RA, independent of its direct impact on muscle health [346]. Furthermore, it has been hypothesised that MSTN could impede irisin biosynthesis, thereby promoting adipose tissue accumulation while concurrently reducing muscle mass, contributing to the development of SO [386].

#### 2.6.2. Irisin

Irisin is a peroxisome proliferator-activated receptor γ coactivator-1α (PGC-1α)-dependent myokine that is released into the bloodstream by cleavage of the type III fibronectin domain (FNDC5) protein-triggered muscle contraction. This release leads to browning and regulation of thermogenesis in WAT [402,403]. Irisin, a myokine originally thought to be exclusively produced by skeletal muscle, has recently been found to be released from adipose tissue as well [404] and in smaller amounts from the liver, bone, testes, pancreas, brain, spleen, heart, and stomach [405,406,407]. At first, interest in irisin was due to its ability as a fat browning inducer and thermogenesis regulator [402,403]; however, further research showed its multipotentiality and its influence on the nervous, cardiac, and musculoskeletal systems. Moreover, its influence on the regulation of energy metabolism and its anti-inflammatory and antioxidative actions have been widely described [408,409,410,411]. Irisin affects target cells by interacting with membrane integrins αV/β5, responsible for cell-to-cell and cell-to-ECM interactions, thus playing an important role in cell activation, proliferation, adhesion, and migration [412].

Irisin has been shown to stimulate the differentiation and development of osteoblasts [409]. Osteoid formation increases osteoblasts while reducing osteoclasts, which is important in bone formation [407,409]. Colaianni et al. [407] showed that irisin affects bone formation in mice by inducing mRNA expression of early osteoblastic differentiation genes, including marrow-activating transcription factor 4 (Atf4), runt-related transcription factor-2 (Runx2), and Sp7 transcription factor (Sp7), which consequently launches a global osteogenesis program. Work by Qiao et al. [413] indicated that irisin promotes osteoblast proliferation, differentiation and mineralisation via p38 MAPK and ERK signalling pathways. Irisin increases the strength of cortical bone and its resistance to bending and torsion by increasing bone mass and improving bone density, length, thickness, periosteal perimeter, and geometry [407,409,413]. However, the anabolic effect of irisin action is specific to long cortical bones and not trabecular bones, where irisin action was not observed [407]. Irisin has also been shown to strengthen the structural support that the subchondral bone provides to cartilage [409]. Irisin exerts an anti-apoptotic effect on osteocytes by increasing the expression of Atf4 [414].

Furthermore, irisin stimulates chondrocyte proliferation, reducing the secretion of inflammatory factors and MMP while increasing the expression of tissue inhibitor of metalloproteinases (TIMP) in these cells [409]. Irisin also reduces the differentiation into osteoblasts in human osteoarthritic chondrocytes (hOAC) collected from OA patients [415], inhibits chondrocyte apoptosis, and increases the stability of the surrounding ECM [409]. The chondrogenic effects of irisin are mediated by the MAPK-NFκB pathway, including inhibition of p38, AKT, and JNK phosphorylation, but not ERK [415]. In a mouse model of OA, irisin exerted a chondroprotective effect by inhibiting inflammation-induced oxidative stress, promoting its biogenesis, preventing mitochondrial fusion and mitophagy, and regulating autophagy and apoptosis for survival [416].

In myotubes, irisin induces the expression of pro-myogenic genes, increases myogenic differentiation, and promotes myoblast fusion [417,418]. The administration of exogenous irisin improves regeneration, induces hypertrophy, and reduces protein degradation by activating satellite cells and increasing protein synthesis in mice [418]. Irisin also has an anti-atrophic effect on C2C12 myotubes treated with dexamethasone, a recognised inducer of muscle atrophy, by inhibiting FoxO-dependent ubiquitin-proteasome overactivity [419].

In animal experiments, inhibiting MSTN caused an increase in irisin levels [420]. Irisin has been previously associated with a reduction in adipose tissue mass and an enhancement in insulin sensitivity [421,422].

Recent research utilising rats with experimental arthritis showed that irisin has therapeutic potential due to its anti-inflammatory and antioxidant actions [423]. Furthermore, RA patients had substantially reduced irisin levels in their serum when compared to healthy controls [342,349,350], and irisin levels were significantly inversely correlated with disease activity and disability in RA patients [342,349] (Table 5). Low serum irisin levels were also associated with the presence of vertebral fractures in RA-positive women [350]. Interestingly, poor sleep quality in RA patients may be linked to decreased serum irisin levels, suggesting a possible association between sleep impairment and irisin levels in healthy controls [342].

## 3. Conclusions

Experimental and clinical evidence demonstrates the significance of adipokines and adipomyokines such as irisin and MSTN in RA. These molecules can contribute to inflammation, immune dysregulation, joint destruction, and metabolic disturbances in this disease. Emerging research highlights the intricate interaction between adipose tissue and skeletal muscle, further implicating their role in RA.

Adipokines have shown promise as biomarkers in RA, providing valuable information regarding disease activity, prognosis, and response to treatment. Several adipokines, including adiponectin, leptin, resistin, and visfatin, have been investigated as potential biomarkers for RA diagnosis and disease monitoring. Dysregulation of these adipokines in serum or SF is correlated with disease severity, joint damage, and systemic manifestations in RA patients. Furthermore, elevated levels of MSTN might function as valuable biomarkers for the detection of individuals susceptible to the development of rheumatoid cachexia and myopenia.

The identification of adipokines as potential therapeutic targets also provides new opportunities for the treatment of RA. It may be possible to modulate the levels and functions of pro-inflammatory adipokines while simultaneously enhancing the production or effectiveness of anti-inflammatory adipokines. Focusing on MSTN could potentially alleviate muscle wasting, enhance metabolic regulation, and regulate the inflammatory environment within RA. Nonetheless, certain challenges need to be addressed to successfully translate adipokine-based therapies into clinical practice, such as comprehending the intricate nature of the network formed by these molecules, accounting for patient heterogeneity, and developing techniques for targeted delivery into specific tissues. In order to optimise combination therapies, it is essential to grasp how adipokines interact with current treatments for RA. By incorporating adipokine profiling into clinical strategies, early detection can be improved, treatment decisions can be better guided, and disease activity in RA can be efficiently monitored.

In summary, adipokines and adipomyokines play a crucial role in the pathogenesis of RA and have the potential as biomarkers for disease diagnosis, monitoring, and prognosis. Further research on adipokines and adipomyokines will aid in the development of personalised and targeted therapeutic strategies, ultimately improving outcomes and patient care in RA.

## Figures and Tables

**Table 1:** Association of leptin and rheumatoid arthritis in humans.

Reference	Subjects	Results/Outcomes
Anders et al. (1999) [209]	58 RA patients 16 controls without RA	No differences were observed in serum leptin levels between RA patients and healthy controls. No correlations existed between serum leptin levels and DAS.
Bokarewa et al. (2003) [198]	76 RA patients 34 controls without RA	Plasma levels in RA patients were significantly greater than in controls. Leptin levels in RA patients were significantly greater in plasma than in SF samples,
Popa et al. (2005) [210]	31 RA patients 19 controls without RA	No differences between RA patients and healthy controls in plasma leptin levels were observed. In RA patients, plasma leptin levels were inversely correlated with CRP and IL6 levels.
Hizmetli et al. (2005) [211]	41 RA patients 25 controls without RA	No significant difference between RA patients and the control group was observed in plasma leptin levels. No correlation was observed between serum leptin level and disease duration, ESR, or CRP in RA patients.
Otero et al. (2006) [200]	31 RA patients 18 controls without RA	Leptin serum levels in RA patients were significantly greater than in controls. A positive correlation was observed between serum leptin and CRP levels.
Gunaydin et al. (2006) [212]	50 RA patients 34 controls without RA	Serum leptin levels were greater in RA patients than in controls. No correlations existed between serum leptin levels and disease duration, swollen and tender joint counts, DAS28, CRP, ESR, and serum TNFα levels.
Lee et al. (2007) [213]	50 RA patients	Serum leptin levels were greater in RA patients with DAS28 > 3.2. There was a positive correlation between serum leptin levels and DAS28 and CRP.
Wislowska et al. (2007) [214]	30 RA patients, 30 OA patients	There were no significant differences in serum leptin levels between RA and OA patients. No correlation existed between serum leptin level and disease duration, duration of morning stiffness, and DAS28 in RA patients.
Seven et al. (2009) [215]	20 RA patients 25 controls without RA	Serum and SF leptin levels were significantly greater in RA patients than in the control group. RA patients with moderate disease activity (DAS > 2.7) had significantly greater leptin levels than those with low disease activity (DAS < 2.7)
Canoruç et al. (2009) [216]	52 RA patients 52 controls without RA	Plasma leptin levels in RA patients were significantly greater than in controls. Plasma leptin level significantly correlated with ESR and swollen and tender joint count.
Targonska-Stepniak et al. (2010) [217]	80 RA patients	A positive correlation existed between leptin levels and the DAS28.
Ismail et al. (2011) [218]	40 RA patients 30 controls without RA	Serum leptin levels were greater in RA patients than in controls. A positive correlation was observed between serum leptin levels and the DAS28.
Yoshino et al. (2011) [219]	141 RA patients 146 controls without RA	A positive correlation was observed between serum leptin and CRP levels.
Olama et al. (2012) [203]	40 RA patients 30 controls without RA	Leptin serum levels in RA patients were significantly greater than in controls. Serum leptin level and synovial/serum leptin ratio significantly correlated with the RA duration, DAS28, ESR, CRP, TNFα, and IL-6. The serum leptin and the synovial/serum leptin ratio were significantly greater in women than in men and in erosive RA than non-erosive RA. The synovial/serum leptin ratio was positively associated with erosion in patients with RA.
Allam et al. (2012) [220]	37 RA patients 34 controls without RA	Serum leptin levels were greater in RA patients than in controls. There was no correlation between serum leptin level and disease duration, duration of morning stiffness, VAS, number of swollen and tender joints, DAS28, ESR or CRP in patients with RA.
Mirfeizi et al. (2014) [221]	54 RA patients (29 with erosion, 25 without erosion)	The two groups had no significant differences in mean serum leptin levels.
Abdalla et al. (2014) [222]	60 RA patients 30 controls without RA	Serum leptin levels were greater in RA patients than in controls. No correlation between leptin levels and the DAS28.
Bustos Rivera-Bahena et al. (2015) [223]	121 RA patients	A positive correlation existed between leptin serum levels and disease activity.
Oner et al. (2015) [224]	106 RA patients 52 healthy controls 37 OA patients	There were no differences in serum leptin levels between groups. No correlation was observed between serum leptin levels and DAS28.
Dervisevic et al. (2018) [205]	55 RA patients, 25 controls without RA	Serum leptin levels were greater in RA patients than in controls. A positive correlation existed between leptin serum levels and DAS28.
Wang et al. (2018) [204]	54 RA patients, 46 controls without RA	Serum leptin levels were greater in RA patients than in controls.
Chihara et al. (2020) [167]	136 RA patients 78 controls without RA	Serum leptin levels were greater in RA patients than in controls.
Rodriguez et al. (2021) [225]	51 eRA patients 51 controls without RA	Serum leptin levels were significantly greater in patients with eRA than in healthy controls.

RA = rheumatoid arthritis; eRA= early RA; OA = osteoarthritis; CRP = C-reactive protein; TNFα = tumour necrosis factor-α; IL-6 = Interleukin-6; ESR = erythrocyte sedimentation rate; DAS = Disease Activity Score; DAS28 = DAS28 = Disease Activity Score 28; VAS = visual analogue scale, SF = synovial fluid.

**Table 2:** Association of adiponectin and rheumatoid arthritis in humans.

Reference	Subjects	Results/Outcomes
Senolt et al. (2006) [258]	20 RA patients, 21 OA patients 23 healthy controls	Serum adiponectin levels were significantly greater in RA patients than in healthy controls and comparable with OA patients. SF adiponectin was significantly greater in RA than in OA patients and healthy controls.
Otero et al. (2006) [200]	31 RA patients 18 controls without RA	Serum adiponectin levels in RA patients were significantly greater than in controls. A positive correlation was observed between serum adiponectin and CRP levels.
Laurberg et al. (2009) [263]	114 RA patients, 35 OA patients 45 healthy controls	RA patients had greater plasma adiponectin concentration than healthy controls but lower plasma adiponectin than OA patients.
Ebina et al. (2009) [265]	90 RA patients 42 controls without RA	RA severity was assessed by examining joint destruction on radiographs. Serum adiponectin levels were significantly greater in severe RA patients when compared to mild and control groups, suggesting increased joint destruction is linked to hyperadiponectinaemia in RA patients.
Popa et al. (2009) [264]	58 RA patients 58 controls without RA	Serum adiponectin levels were significantly greater in all RA patients than in controls.
Rho et al. (2010) [202]	167 RA patients 91 controls without RA	Serum adiponectin levels were significantly greater in all RA patients than in controls.
Ozgen et al. (2010) [262]	56RA patients 29 controls without RA	Serum adiponectin levels in RA patients were significantly grater than in controls. A positive correlation was observed between serum adiponectin and DAS28.
Alkady et al. (2011) [260]	70 RA patients 30 controls without RA	The serum levels of adiponectin were significantly greater in RA patients compared to controls, and RA patients with active disease had significantly greater adiponectin levels than those in remission. Serum and SF adiponectin levels positively correlated with the duration of disease, ESR, CRP, and DAS28 in RA patients with active disease.
Klein-Wieringa et al. (2011) [271]	253 RA patients	A positive correlation existed between serum levels of adiponectin and radiographic progression of RA over 4 years.
Giles et al. (2011) [272]	152 RA patients	A positive correlation existed between serum adiponectin levels and erosive joint destruction.
Ozgen et al. (2010) [262]	56 RA patients 29 controls without RA	Serum adiponectin was significantly greater in RA patients than in healthy controls.
Yoshino et al. (2011) [219]	141 RA patients 146 controls without RA	Female, but not male, RA patients had significantly greater serum adiponectin concentrations than controls. Serum adiponectin levels were negatively associated with CRP levels.
Kang et al. (2013) [273]	192 RA patients	There was a significant correlation between serum adiponectin levels and ESR.
Meyer et al. (2013) [274]	632 RA patients	A positive association was observed between serum adiponectin levels and early radiographic RA progression.
Toussirot et al. (2013) [275]	70 RA patients 51 controls without RA	Serum adiponectin levels were significantly greater in RA patients than in controls. In RA patients, HMW/total adiponectin correlated with the DAS28.
Bustos Rivera-Bahena et al. (2015) [223]	121 RA patients	No correlation existed between serum adiponectin and DAS28. A negative correlation existed between serum adiponectin and TNFα. A positive correlation existed between serum adiponectin and IL-11β.
Chennareddy et al. (2016) [268]	43 RA patients 25 controls without RA	Serum levels of adiponectin were significantly greater in RA patients when compared to the control group. There was no correlation with an erosive and non-erosive disease, disease duration, BMI, waist–hip ratio, and DAS28.
Khajoei et al. (2019) [261]	90 RA patients 30 controls without RA	Serum adiponectin levels were greater in all RA patients than in controls. There was a positive correlation between serum adiponectin levels and DAS28 and ESR.
Zhang et al. (2020) [269]	3693 obese subjects followed for up to 29 years.	High serum adiponectin levels at baseline were a significant risk factor for RA in the cohort of subjects with obesity.
Vasileiadis et al. (2021) [232]	70 RA patients	Both total and HMW adiponectin were positively associated with DAS28 and CRP in RA patients.
Baker et al. (2022) [276]	2583 RA patients	Serum adiponectin levels were strongly associated with radiographic damage, seropositivity, longer disease duration, and circulating inflammatory cytokines.

RA = rheumatoid arthritis; OA = osteoarthritis; CRP = C-reactive protein; TNFα = tumour necrosis factor-α; ESR = erythrocyte sedimentation rate; DAS28 = Disease Activity Score 28; BMI = body mass index.

**Table 3:** Association of visfatin and rheumatoid arthritis in humans.

Reference	Subjects	Results/Outcomes
Otero et al. (2006) [200]	31 RA patients 18 controls without RA	Serum visfatin levels in RA patients were significantly greater than in controls. There was a positive correlation between serum visfatin and CRP levels.
Matsui et al. (2008) [286]	22 RA patients 17 controls without RA	Serum visfatin levels in RA patients were significantly greater than in controls.
Rho et al. (2010) [202]	167 RA patients 91 controls without RA	A positive correlation existed between visfatin serum levels and TNFα, IL-6, CRP, neutrophil count, MHAQ score, and Larsen score.
Alkady et al. (2011) [260]	70 RA patients 30 controls without RA	Serum levels of visfatin were significantly greater in RA patients than in controls, and RA patients with active disease had significantly greater visfatin levels than those in remission. Serum and SF visfatin levels positively correlated with the duration of the disease, ESR, CRP, and DAS28 in RA patients with active disease.
Klein-Wieringa et al. (2011) [271]	253 RA patients	A positive correlation existed between visfatin serum levels and the radiographic progression of RA for 4 years.
Ozgen et al. (2011) [288]	26 RA patients 29 controls without RA	Serum visfatin levels in RA patients were significantly greater than in controls.
Meyer et al. (2013) [274]	632 RA patients	No association between serum visfatin level and radiographic disease progression was observed.
Khalifa et al. (2013) [301]	60 RA patients 20 controls without RA	There was a significant positive correlation between serum visfatin and ESR, CRP, IL-6, TNFα, DAS-28, and VAS pain score in RA patients. No correlation existed between serum visfatin and age of RA patients, disease duration, or BMI.
El-Hini et al. (2013) [287]	40 RA patients 40 controls without RA	Serum visfatin levels in RA patients were significantly greater than in controls. A positive correlation was observed between serum visfatin and DAS28.
Sglunda et al. (2014) [166]	40 eRA patients, 30 controls without RA	eRA patients had elevated serum visfatin levels compared to healthy controls, which decreased after three months of treatment. Circulating visfatin and visfatin level changes correlated with disease activity and improved over time, with a decrease in visfatin predicting improvement in DAS28.
Mirfeizi et al. (2014) [221]	54 RA patients (29 with and 25 without erosion)	Serum visfatin levels were greater in patients with radiographic joint damage and dependent on the duration of the disease.
Mohammed Ali et al. (2020) [302]	60 RA patients 30 controls without RA	Serum visfatin levels in RA patients were significantly greater than in controls. There was no correlation between serum visfatin levels and the DAS28.

RA = rheumatoid arthritis; eRA = early RA; CRP = C-reactive protein; TNFα = tumour necrosis factor-α; IL-6 = Interleukin-6; ESR = erythrocyte sedimentation rate; DAS28 = Disease Activity Score 28; SF = synovial fluid.

**Table 4:** Association of resistin and rheumatoid arthritis in humans.

Reference	Subjects	Results/Outcomes
Otero et al. (2006) [200]	31 RA patients 18 controls without RA	Serum resistin levels in RA patients were significantly greater than in controls. There was no correlation between serum resistin and CRP level.
Migita et al. (2006) [316]	42 RA patients 38 controls without RA	Serum resistin levels in RA patients were significantly greater than in controls. Serum resistin levels were correlated with RA disease activity markers, CRP, ESR, and TNFα levels.
Forsblad d’Elia et al. (2008) [321]	90 RA patients 30 controls without RA	Serum resistin levels were not significantly different between patients and healthy controls. Serum resistin levels were correlated with IL-1Ra, CRP, TNFα, ICTP, glucocorticosteroids, Larsen score, and inversely with BMD, hip, and TLM.
Rho et al. (2009) [202]	167 RA patients 91 controls without RA	Serum resistin levels were significantly greater in all RA patients than in the control group.
Canoruç et al. (2009) [216]	52 RA patients 52 controls without RA	Plasma resistin levels in RA patients were significantly greater than in controls.
Kassem et al. (2010) [315]	30 RA patients 15 controls without RA	Serum and SF resistin levels in RA patients were significantly greater than in controls and in active RA greater than in non-active patients. A significant correlation existed between serum resistin levels and CRP, ESR, RF, and disease activity. A significant correlation existed between SF resistin levels and CRP, ESR, RF, and synovial leukocytic count.
Yoshino et al. (2011) [219]	141 RA patients 146 controls without RA	Serum resistin levels were not significantly different between patients and healthy controls. A positive correlation was observed between serum resistin and CRP levels.
Alkady et al. (2011) [260]	70 RA patients 30 controls without RA	No significant difference in serum resistin levels was observed between RA patients and controls.
Yoshino et al. (2011) [219]	141 RA patients 146 controls without RA	A positive correlation was observed between serum resistin and CRP levels.
Fadda et al. (2013) [317]	25 RA patients 25 OA patients	Resistin levels were greater in serum and SF of RA patients when compared to OA patients. There was a correlation between serum resistin levels, Larsen score, and total leukocyte count. A correlation existed between SF resistin levels and RF, ACPA, and Larsen score.
Kang et al. (2013) [273]	192 RA patients	A significant correlation existed between serum resistin levels, ESR, CRP, and disease duration.
Hammad et al. (2014) [319]	30 RA patients 30 controls without RA	Serum resistin levels in RA patients were significantly greater than in controls.
Bustos Rivera-Bahena et al. (2015) [223]	121 RA patients	A positive correlation was observed between resistin serum levels and the DAS28.
Vuolteenaho et al. (2022) [322]	99 RA patients	High resistin levels were associated with active inflammatory disease and predicted rapid radiological progression during 5-year follow-up.
Arias-de la Rosa et al. (2022) [206]	150 RA patients 50 controls without RA	Serum resistin levels in RA patients were significantly greater than in controls. A positive correlation was observed between resistin serum levels and the DAS28.

RA = rheumatoid arthritis; OA = osteoarthritis; CRP = C-reactive protein; TNFα = tumour necrosis factor-α; ESR = erythrocyte sedimentation rate; DAS28 = Disease Activity Score 28; ICTP = carboxyterminal cross-linked telopeptide of type I collagen; TLM = total lean mass, RF = rheumatoid factor.

**Table 5:** Association of other selected adipokines and adipomyokines and rheumatoid arthritis in humans.

Reference	Subjects	Results/Outcomes
Ozgen et al. (2010) [262]	56 RA patients 29 controls without RA	Serum vaspin levels were significantly greater in RA patients than in healthy controls.
Wahba et al. (2021) [335]	150 RA patients 150 controls without RA	Serum levels of chemerin and vaspin were greater, while those of apelin and omentin were lower, in RA patients than in healthy controls. A significant positive correlation existed between vaspin serum levels and BMI, DAS28, ESR, CRP, RF, and anti-CCP antibodies.
Senolt et al. (2010) [334]	33 RA patients, 33 OA patients	SF vaspin levels were significantly greater in RA patients than in OA patients. A positive correlation existed between vaspin SF levels and DAS28. SF omentin levels were significantly lower in RA patients than in OA patients.
Ha et al. (2013) [336]	71 RA patients 42 controls without RA	Plasma chemerin levels in RA patients were significantly greater than in controls. There was a positive correlation between plasma chemerin levels and DAS28.
Yamamoto et al. (2014) [337]	56 RA patients, 31 OA patients 417 controls without RA	Serum PGRN levels in RA patients were significantly greater than in controls. SF PGRN levels were significantly greater in RA patients than in OA patients.
Cerezo et al. (2015) [338]	47 RA patients, 42 OA patients 41 controls without RA	Serum PGRN levels correlated with DAS28 and HAQ scores in RA patients. The SF PGRN levels were significantly greater in RA than in healthy controls and OA patients.
Chen et al. (2016) [339]	80 RA patients, 60 controls without RA	Serum PGRN levels in RA patients were significantly greater than in controls. A significant positive correlation existed between serum PGRN and DAS28, ESR, and CRP in RA patients.
Fouad et al. (2019) [340]	52 RA patients, 19 controls without RA	Serum PGRN levels in RA patients were significantly greater than in controls. A significant positive correlation existed between serum PGRN, DAS28, and ESR in RA patients.
Kvlividze et al. 2019) [341]	110 RA patients, 60 controls without RA	RA patients with severe disease had greater serum nesfatin-1 levels, positively correlated with greater CRP and ESR concentrations.
Gamal et al. (2020) [342]	58 RA patients, 30 controls without RA	Serum irisin levels were significantly lower in RA patients than in controls. Serum irisin levels in RA-poor sleepers were significantly lower than in RA-good sleepers. Serum irisin levels in RA patients were associated with total Pittsburgh Sleep Quality Index scores. Irisin serum levels of RA patients were negatively associated with disease duration, morning stiffness duration, and DAS28.
Gonzalez-Ponce et al. (2021) [343]	210 RA patients	A positive correlation was found between chemerin and DAS 28, CRP, and swollen joints counts.
Vazquez-Villegas et al. (2021) [344]	82 RA patients	Elevated chemerin levels were associated with functional disability in RA.
Zhang et al. (2021) [345]	40 RA patients, 15 controls without RA	Nesfatin-1 levels in the synovium were significantly greater in RA patients than in controls. Nesfatin-1 levels positively correlated with IL-1β and TNFα levels in the synovium of patients with RA.
Murillo-Saich et al. (2021) [346]	84 RA patients, 127 controls without RA	MSTN serum levels were significantly greater in the RA group than in the controls. Greater MSTN levels correlated with low skeletal muscle mass index. A positive correlation existed between MSTN serum levels and DAS28, CRP and ESR.
Lin et al. (2022) [347]	344 RA patients, 118 controls without RA	In a prospective cohort of consecutive RA patients with a one-year follow-up, it was found that RA patients had greater serum MSTN levels at baseline when compared to healthy controls. Elevated serum MSTN and myopenia are risk factors for radiographic progression in RA. RA patients with elevated MSTN overlapping myopenia had the highest levels of DAS28, SDAI, CDAI, and HAQ-DI at 3, 6, and 12 months, with the lowest proportion of CDAI remission at 3 months, 9 months, and 12 months. They also had the highest proportion of physical dysfunction at 3, 9, and 12 months.
Gonzalez-Ponce et al. (2022) [348]	161 RA patients, 72 controls without RA	MSTN serum levels were significantly greater in the RA group than in the controls. Elevated MSTN levels in RA patients increased the risk of cachexia.
Soliman et al. (2022) [349]	60 RA patients, 30 controls without RA	Serum irisin levels were significantly lower in RA patients than in controls. Serum irisin levels in RA patients negatively correlated with DAS28, CRP, ESR, and HAQ-DI.
Gamez-Nava et al. (2022) [350]	148 RA patients, 97 controls without RA	Serum irisin levels were significantly lower in RA patients than in controls. Low serum irisin levels were associated with the presence of vertebral fractures in female patients with RA.
Arias-de la Rosa et al. (2022) [206]	150 RA patients 50 controls without RA	Serum omentin and vaspin levels in RA patients were significantly higher than in controls. A positive correlation was observed between omentin serum levels and the DAS28.

RA = rheumatoid arthritis; OA = osteoarthritis; CRP = C-reactive protein; TNFα = tumour necrosis factor-α; IL-6 = Interleukin-6; ESR = erythrocyte sedimentation rate; DAS28 = Disease Activity Score 28; PGRN = progranulin; MSTN = myostatin; CDAI = clinical disease activity index; SDAI = simplified disease activity index; HAQ-DI = health assessment questionnaire–disability index; SF = synovial fluid.

## Data Availability

Not applicable.
